# Supplementary material for: Influence of the use of an adhesive connection on the joint strength of modular hip endoprostheses
Source: PLoS One. 2024 Nov 18;19(11):e0313964. doi: 10.1371/journal.pone.0313964 (PMC11573162; doi:10.1371/journal.pone.0313964)
Supplement: S1 Table — Overview over all 28 taper pairs with their push-out force and taper mismatch (angular difference). The colors indicate which taper was sandblasted: grey: outer taper sandblasted, light blue: trunnion sandblasted, dark blue: both sandblasted. (DOCX) [file pone.0313964.s001.docx]

| **Taper group - Taper Pair** | **Push-Out Force** | **Taper mismatch** |
| --- | --- | --- |
| Ti 1-1 | 0,393 | -0.1844 |
| Ti 1-2 | 1,210 | -0.2356 |
| Ti 1-3 | 0,804 | -0.192 |
| Ti 1-4 | 1,385 | -0.18 |
| Ti 2-1 | 4,402 | -0.159 |
| Ti 2-2 | 0,751 | -0.1866 |
| Ti 2-3 | 2,177 | -0.1848 |
| Ti 2-4 | 2,301 | -0.1676 |
| Ti 3-1 | 0,645 | -0.1682 |
| Ti 3-2 | 0,672 | -0.1816 |
| Ti 3-3 | 0,790 | -0.2086 |
| Ti 3-4 | 1,019 | -0.2004 |
| Ti 4-1 | 7,127 | -0.1208 |
| Ti 4-2 | 0,752 | -0.1216 |
| Ti 4-3 | 0,387 | -0.1504 |
| Ti 4-4 | 0,865 | -0.1536 |
| CoCr 5-1 | 6,153 | -0.2218 |
| CoCr 5-2 | 2,074 | -0.2372 |
| CoCr 5-3 | 1,250 | -0.1702 |
| CoCr 5-4 | 0,914 | -0.17 |
| CoCr 6-1 | 0,710 | -0.1424 |
| CoCr 6-2 | 0,627 | -0.14 |
| CoCr 6-3 | 0,605 | -0.150 |
| CoCr 6-4 | 0,285 | -0.1834 |
| CoCr 7-1 | 5,108 | -0.2098 |
| CoCr 7-2 | 0,645 | -0.1658 |
| CoCr 7-3 | 0,398 | -0.1522 |
| CoCr 7-4 | 0,349 | -0.3688 |

**S1 Table**: Overview over all 28 taper pairs with their push-out force and taper mismatch (angular difference). The colors indicate which taper was sandblasted: grey: outer taper sandblasted, light blue: trunnion sandblasted, dark blue: both sandblasted
