# Supplementary material for: Influence of the use of an adhesive connection on the joint strength of modular hip endoprostheses
Source: PLoS One. 2024 Nov 18;19(11):e0313964. doi: 10.1371/journal.pone.0313964 (PMC11573162; doi:10.1371/journal.pone.0313964)
Supplement: Attachment — Submitted filename: PONE-D-24-03532_review.pdf [file pone.0313964.s004.pdf]

The paper presents an interesting approach to secure taper connections in total hip arthroplasty against fretting and fretting-corrosion, respectively, via adhesive bonding of the connection. To do so, taper connections were fabricated, adhesively bonded and, subsequently, the resulting bonding strength was assessed in push-out tests. For inner tapers, a variety of different fabrication parameters was used which resulted in different surface topographies. The different topographies, however, did not influence the resulting bonding strength. In addition, one part or both parts of the taper connections were sandblasted, which influenced the resulting push-out forces.

It was, however, not clearly addressed, which taper connection would benefit from adhesive bonding, since modularity (aside from the better adjustment options) also allows the replacement of individual parts of the endoprosthesis in case of a revision. This would in my opinion be compromised by adhesive bonding unless the bonding could be unglued in case of a revision (?). This should definitely be addressed in the discussion of the study. Furthermore, it remained unclear to me, why so many different combinations processing parameters were used (Table 1 is rather confusing), especially since they did not really influence the resulting bonding strengths. Here, the paper would benefit from reducing the presented magnitude of different taper variations. Maybe the focus could be put on sandblasting vs. turned surfaces and the used materials?

Major revisions are, therefore, necessary before publication of the study.

Some more specific comments are the following:

Introduction:

As already pointed out, it remains unclear, which taper connection should or could be addressed with adhesive bonding approach. In the introduction you refer to studies regarding dual-neck total hip endoprosthesis, but these have widely disappeared from the market, as far as I know. The taper junction you actually tested appears to rather mimic the taper connection between head and stem taper (even though you used a twice as large taper angle).

Material and Methods:

Line 108: The taper angle is given as 5,665° but from Figure 1 it appears that this value refers to the half of the taper angle, which would result in a taper angle > 11°. Please be more accurate here and throughout the manuscript.

Line 114 -125: It is very hard for the reader to understand the combination of the process parameters used for manufacturing the specimens. In line 115 for example you write that two cutting speeds and three feeds were used, which would make six. From the table it becomes clear that not all parameters were combined with each other, but this does not become clear from the text. Also, it does not become clear, which material was used for which parameter combination and whether you produced inner and outer tapers from the two materials. Furthermore, it remains unclear how the different materials were combined for forming the taper connections. Did you just couple Ti-Ti and CoCr-CoCr? If so, why not couple CoCr-Ti as is done in many head-neck taper connections?

Line 180: How was the axial displacement determined (e.g. traverse position, optical measurement…)? I ask because I wonder whether the different inclinations are really caused by the “stiffness” of the connection as you write in line 318ff or if they reflect rather the deformation of the testing setup with increasing load levels.

Results

Table 2: Does the table refer to inner or outer tapers? How are the roughness values for the not sandblasted surfaces?

Figure 3: Does it refer to inner or outer tapers? Please add the information in the figure caption.

The term “Taper mismatch” is used in two different contents, in line 185 ff it refers to the deviation from the targeted value, later in the paper it refers to the difference between inner and outer taper. This is somehow confusing since “taper mismatch” is more commonly used synonymously to taper clearance (i.e. with respect to the difference between inner and outer taper).

Line 201-204: The slight differences in taper end diameters do not necessarily result in a changed taper contact situation but different taper angles do (see image). Please use the difference between taper angles in order to characterize the contact (distal or proximal), since using end diameters is very confusing und unusual.

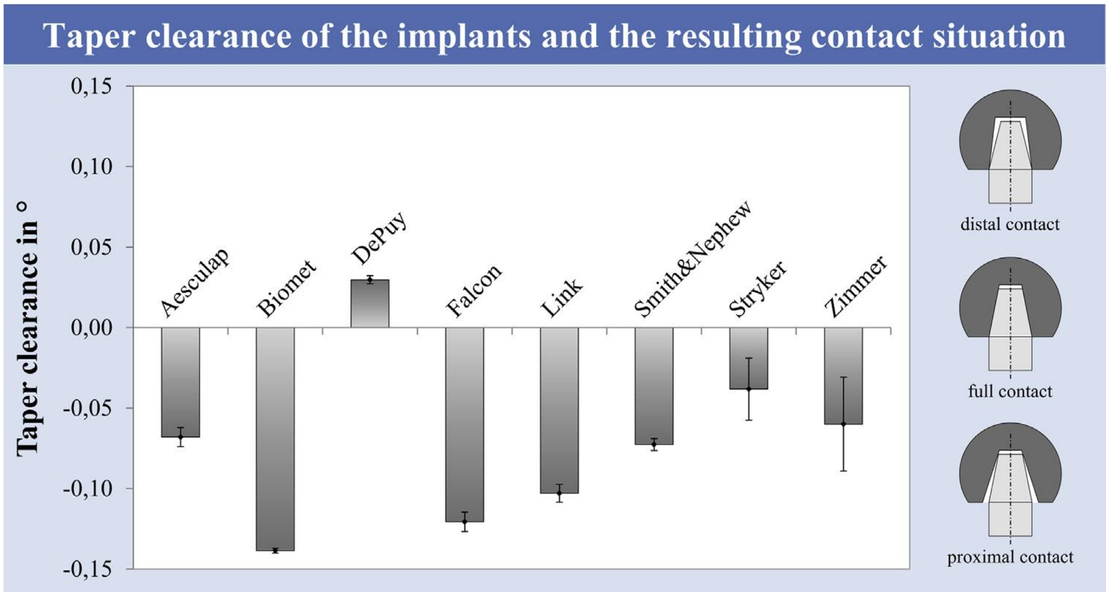

(Mueller et al., J Arthroplasty 2017, 32(10), 3191)

Line 245: Is the correlation statistically significant?

Line 268: If the adhesive is not observed after disconnecting the tapers, where do you think it is gone to? Is it likely that chips of dried adhesive have detached from the surface? If so, this would be a drawback since chips of the glue might act as three-body in the joint articulation or cause tissue reactions. This could be addressed in the discussion.

Discussion

Line 282f: See comment above (line 201-204). Please refer to the taper angle mismatch if discussing the contact situation.
