# Supplementary material for: Influence of the use of an adhesive connection on the joint strength of modular hip endoprostheses
Source: PLoS One. 2024 Nov 18;19(11):e0313964. doi: 10.1371/journal.pone.0313964 (PMC11573162; doi:10.1371/journal.pone.0313964)
Supplement: Attachment — Submitted filename: PONE-D-24-03532_R1.Reviewed.pdf [file pone.0313964.s006.pdf]

PLOS ONE

Influence of the use of an adhesive connection on the joint strength of modular hip endoprostheses

--Manuscript Draft--

**Manuscript Number:**	PONE-D-24-03532R1
**Article Type:**	Research Article
**Full Title:**	Influence of the use of an adhesive connection on the joint strength of modular hip endoprostheses
**Short Title:**	Adhesive connection of modular hip endoprostheses
**Corresponding Author:**	Bastian Welke<br>Hannover Medical School: Medizinische Hochschule Hannover<br>Hannover, GERMANY
**Keywords:**	modular hip endoprostheses, adhesive connection, junction strength, taper junction
**Abstract:**	Introduction<br>Modular hip implants enables a more precise adaptation of the prosthesis to the patient's anatomy. However, they also carry the risk of increased revision rates due to micromotion at the taper junction. In order to minimize this risk, one potential solution is to establish an adhesive bond between the metal taper junctions. Load-stable bonding techniques, already successfully employed in dentistry for connecting materials such as metals and ceramics or different alloys, offer a promising approach. Nevertheless, the bond strength of tapered adhesive bonds in modular hip implants has not been investigated to date.<br>Materials and Methods<br>Twenty-eight tapered junctions, consisting of a taper (female taper) and a trunnion (male taper) were turned using TiAl6V4 ELI (n = 16) and CoCr28Mo6 (n = 12). The process parameters cutting speed (vc = 50 m/min or 100 m/min) and feed (f = 0.1 mm, 0.05 mm or 0.2 mm) were varied for the trunnions. For each set of process parameters, one trunnion and one taper were additionally subjected to sandblasting. To investigate the effect of geometry, angular mismatch in the samples were measured. The taper pairs were bonded with a biocompatible adhesive, and push-out tests were subsequently performed.<br>Results<br>The push-out forces generated from the taper connections where both tapers were sandblasted showed a mean push-out force of 5.70 kN. For the samples with only the trunnion sandblasted, the mean force was 0.58 kN, while for the samples with only taper sandblasted the mean push-out force was 1.32 kN. When neither of the tapers was sandblasted the mean push-out force was 0.91 kN. No significant effect of the process parameters on the push-out force was observed. Only the reduced valley depth Svk showed a slight correlation for the CoCr28Mo6 samples ($R^2 = 0.54$). The taper pairs with taper mismatch (between trunnion and taper) greater than $\|0.1°\|$ did not show lower push-out forces than the specimens with lower taper mismatch.<br>Conclusions<br>Sandblasted and adhesive-bonded tapered connections represent a viable suitable alternative for modular hip implant connections. Slight differences in taper geometry do not result in reduced push-out forces and are compensated by the adhesive. In mechanically joined tapers these differences can lead to higher wear rates. Further investigation under realistic test conditions is necessary to assess long-term suitability.
**Order of Authors:**	Ann-Kathrin Einfeldt
	Beate Legutko
	Philipp-Cornelius Pott, Dr.
	Benjamin Bergmann, Dr.
	Berend Denkena, Prof.
	Christof Hurschler, Prof.

Powered by Editorial Manager® and ProduXion Manager® from Aries Systems Corporation

	Bastian Welke
**Response to Reviewers:**	Dear Dr. Antonio Riveiro Rodríguez,<br>Thanks a lot for the opportunity to revise our paper in order to resubmit it to PLOS ONE. We have worked on all comments and have implemented all of the suggestions and points of criticism. The present response is done in a stepwise way regarding the reviewer's comments (italic). Corresponding changes and additions have been highlighted in yellow in the "manuscript with track changes".<br>Reviewer #1: The paper presents an interesting approach to secure taper connections in total hip arthroplasty against fretting and fretting-corrosion, respectively, via adhesive bonding of the connection. To do so, taper connections were fabricated, adhesively bonded and, subsequently, the resulting bonding strength was assessed in push-out tests. For inner tapers, a variety of different fabrication parameters was used which resulted in different surface topographies. The different topographies, however, did not influence the resulting bonding strength. In addition, one part or both parts of the taper connections were sandblasted, which influenced the resulting push-out forces.<br><br>It was, however, not clearly addressed, which taper connection would benefit from adhesive bonding, since modularity (aside from the better adjustment options) also allows the replacement of individual parts of the endoprosthesis in case of a revision. This would in my opinion be compromised by adhesive bonding unless the bonding could be unglued in case of a revision (?). This should definitely be addressed in the discussion of the study.<br>Thank you very much for this important note. Our paper in fact focusses on the connection between the shank and a modular taper. Your comment regarding the replacement of the individual parts is really valuable and we added a section in the discussion (L347-350 in the revised manuscript).<br><br>Furthermore, it remained unclear to me, why so many different combinations processing parameters were used (Table 1 is rather confusing), especially since they did not really influence the resulting bonding strengths. Here, the paper would benefit from reducing the presented magnitude of different taper variations. Maybe the focus could be put on sandblasting vs. turned surfaces and the used materials?<br>Thank you very much for this valuable comment. We agree that the table was quite confusing. We have simplified the table and described the sample groups in the text more clearly (L130-140). We nonetheless think that investigating different process parameters provides valuable information and that interestingly the different process parameters did not have an influence on the bonding strength which we did not anticipate.<br>Major revisions are, therefore, necessary before publication of the study.<br><br>Some more specific comments are the following:<br><br>Introduction:<br><br>As already pointed out, it remains unclear, which taper connection should or could be addressed with adhesive bonding approach. In the introduction you refer to studies regarding dual-neck total hip endoprosthesis, but these have widely disappeared from the market, as far as I know. The taper junction you actually tested appears to rather mimic the taper connection between head and stem taper (even though you used a twice as large taper angle).<br><br>Thank you very much for this note. As stated above, the paper focusses on the connection between the shank and a modular taper. We are aware of the disappearance of dual-taper junctions from the market because of the high revision rates. The motivation of this study was to investigate a possible improvement of the taper junctions which may allow dual-taper junctions to be safely used again, and in particular to exploit the flexibility in aligning the joint that they can provide in achieving an anatomically aligned joint reconstruction. We have supplemented some parts of the introduction to make this clearer to the reader.<br><br>Material and Methods:<br><br>Line 108: The taper angle is given as 5,665° but from Figure 1 it appears that this value refers to the half of the taper angle, which would result in a taper angle > 11°.

Please be more accurate here and throughout the manuscript.
Thank you very much for this valuable comment. We have adjusted this accordingly so that the taper angle now has the correct value in the whole manuscript (L115-116).
The reviewer is absolutely right that the taper angle is >11°. We chose this larger angle to compare the results from this study with our preliminary study (18). In the preliminary study, we investigated the influence of the surface topography on the push-out force. The larger angle represents an unfavorable situation for the connection, both for the mechanical and for the bonded connection. We intentionally chose this in our project. Due to the larger taper angle, the average push-out force was only 1.8kN. In the current study, the same bonded taper junction achieved forces three times as high. In our view, this shows the potential of the bond.
Nevertheless, we are currently conducting a study in which we are investigating the cone connection with the taper angle of 12/14.

Line 114 -125: It is very hard for the reader to understand the combination of the process parameters used for manufacturing the specimens. In line 115 for example you write that two cutting speeds and three feeds were used, which would make six. From the table it becomes clear that not all parameters were combined with each other, but this does not become clear from the text. Also, it does not become clear, which material was used for which parameter combination and whether you produced inner and outer tapers from the two materials. Furthermore, it remains unclear how the different materials were combined for forming the taper connections. Did you just couple Ti-Ti and CoCr-CoCr? If so, why not couple CoCr-Ti as is done in many head-neck taper connections?
Thank you very much for this valuable comment. As we mentioned a previous comment, we have simplified the table and described the groups in the text more precisely (L130-140). Due to delivery bottlenecks in CoCr we did not have enough material to also test CoCr-Ti, and we will investigate this material pairing in further investigations. We have added a sentence in this regard to the discussion (L348-350).

Line 180: How was the axial displacement determined (e.g. traverse position, optical measurement…)? I ask because I wonder whether the different inclinations are really caused by the “stiffness” of the connection as you write in line 318ff or if they reflect rather the deformation of the testing setup with increasing load levels.
Thank you for this important observation. We in fact only recorded the displacement of the actuator of the materials testing machine. The recorded displacement was thus not corrected in the first version of the manuscript. In order to determine the influence of the setup on the measured displacement, we once again carried out tests using only the setup without samples. The upper stamp pressed directly on the lower structure. We determined a stiffness of 39.111 N/mm for the linear range. We then used this value to correct the measured displacement. In the manuscript, we replaced the diagram accordingly and added the following part (L274-275):
“The measured displacement of the testing machine was subsequently corrected for the stiffness of the setup.”
We have also added this description to the caption of Figure 6.
Since it might be interesting for the reviewer, we show here the figure of how the graphs have changed after the stiffness correction

Results

Table 2: Does the table refer to inner or outer tapers?
Thank you very much for this important observation. It was the trunnion, and we have added this to the table heading.
How are the roughness values for the not sandblasted surfaces?
Thank you very much for this comment. We added the values for the not sandblasted surfaces into the table.

Figure 3: Does it refer to inner or outer tapers? Please add the information in the figure caption.
Thank you very much again. Here as above it was the trunnion. We have added this to the figure heading.

The term “Taper mismatch” is used in two different contents, in line 185 ff it refers to the deviation from the targeted value, later in the paper it refers to the difference

Powered by Editorial Manager® and ProduXion Manager® from Aries Systems Corporation

between inner and outer taper. This is somehow confusing since “taper mismatch” is more commonly used synonymously to taper clearance (i.e. with respect to the difference between inner and outer taper).
Thank you very much for this important comment. We have adapted the terms throughout the manuscript. We now use ‘nominal value’ instead of ‘target’. And we now only use mismatch to refer to the difference between trunnion and taper.

Line 201-204: The slight differences in taper end diameters do not necessarily result in a changed taper contact situation but different taper angles do (see f.ex. Fig. 8 in Mueller et al., J Arthroplasty 2017, 32(10), 3191). Please use the difference between taper angles in order to characterize the contact (distal or proximal), since using end diameters is very confusing und unusual.
We agree with the reviewer in this regard, we have referred to taper-mismatch to describe the difference in angle between the taper and trunnion and removed the first sentence referring to the end-diameters.
We added the following sentence to the manuscript (L211-214):
“All 28 taper pairs exhibit a negative taper mismatch (trunnion angle < taper angle) and therefore a proximal contact. All taper pairs show a taper mismatch greater than |0.1°| with a mean of -0.182 ± 0.047° (range -0.012° to -0.369°) (table in supplemental material).”

Line 245: Is the correlation statistically significant?
We have added the p-value for the correlations that are significant to the text (L259).

Line 268: If the adhesive is not observed after disconnecting the tapers, where do you think it is gone to? Is it likely that chips of dried adhesive have detached from the surface? If so, this would be a drawback since chips of the glue might act as three-body in the joint articulation or cause tissue reactions. This could be addressed in the discussion.
Thank you very much for this comment. The visual analysis showed that the adhesive adheres only to the sandblasted surfaces and not to the turned ones. So, in the case of sandblasting no chips of glue detached from the sandblasted surface. It will be important to investigate the system under dynamic loading to investigate if particles of dried adhesive are generated and escape into the joint.

Discussion

Line 282f: See comment above (line 201-204). Please refer to the taper angle mismatch if discussing the contact situation.
We agree with the reviewer as above, and therefore we changed the wording and removed the first sentence referring to the end-diameters (L282 first submission of the manuscript).

Reviewer #2: This manuscript investigates the use of an adhesive and sandblasting on the potential stability of total hip head-neck modular junctions. The study is well-designed. The main criticisms focus on details in the manuscript that overstate the significance of how this study relates to hip modular junctions since the current application is still far removed, and the discussion of previous literature. Most suggestions are minor. In addition, please edit throughout for written English.

Abstract
The introduction focuses on micromotion as a failure mode. There is a disconnect between these statements and the paper's methods where push-out tests are used. No explanation is given for the disconnect. Please edit accordingly.
Thank you for your comment, in response to this and the authors further comments regarding the introduction, we have significantly edited and reformulated the introduction to address this point.

Introduction
Line 56: citation missing
Thank you very much for the noticing this, we have added an appropriate citation (L55).

Powered by Editorial Manager® and ProduXion Manager® from Aries Systems Corporation

	Lines 65-66: Reference [3] is an investigation of bi-modular implants which have more than 2 parts, and is less relevant to the current study. Include more references here to make this point, there are plenty of investigations in the literature. Similarly, for the next sentence, the titanium neck adapter was actually TMZF, a different Ti-alloy than what is usually used, so the sentences are misleading.<br>The literature cited was chosen to generally illustrate the failure of a very promising technology, for hip replacement, bi-modular implants, and our relatively simple study investigating the effect of adhesive bonding on taper junction push-out force. We have attempted to address the reviewers well taken comments on the relevance of the cited literature and our study in significant revisions we have made to the introduction (as suggested by the reviewer below).<br><br>Lines 81-82: This is not a relevant finding for the introduction. Taper connections of hip implants on the market have an average angular mismatch much smaller, on the order of 1 minute (.0167 degrees), so 0.096 would already be an outlier and is too large to be clinically relevant.<br>Thank you for this important comment, which we will attempt to address. It is true that taper mismatch of common stem and head tapers has recently been reported to range from about 0,025' to -0,14' (Mueller et al., 2017), which indeed is significantly less than the 0.096° (5.76') we chose. Nonetheless, positive values that are significantly higher (for example 0.076° to 0.130° or 4.6' to 7.8' have also been reported), admittedly for an elongated taper design that is no longer available (Haschke et al., 2016). But more to the point, we have however oriented our study on the possible mismatch constellations that could occur in clinical settings as described by Ashkanfar et al. 2017 and Gustafson et al., 2023, who both conservatively estimated realistic possible taper mismatches based on the literature and their own retrievals to be as high as ±12'. The value we chose, about 6' falls well within this range.<br>Suggest an overall re-writing of the literature background about factors that lead to damage in modular THAs. Only a few studies are cited, and they investigate a wide variety of implant types and dimensions that may or may not be relevant to the current study. The paragraphs do not summarize the literature from clinically relevant designs (the bimodular design referenced has been recalled, many studies of angular mismatch reference manufacturing tolerances that, although written in a standard, are much larger than what is actually used by manufacturers to create implants in clinical practice).<br>Thank you for your suggestion, we have significantly reformulated the introduction to better describe our motivation in relation to the literature. The intention of the introduction was not to provide a comprehensive overview of implant types and dimensions in general. Our motivation is based on the failure of bi-modular implants and their subsequent removal for the market, and our exploratory study of the effect of adhesive bonding on the most rudimentary test of junction stability; the push out test, which to our knowledge has not been investigated to date.<br><br>Materials and Methods<br><br>Table 1: The labeling of the samples is confusing. Makes it appears that all inner and outer tapers had the same material (Ti-alloy or CoCrMo alloy)? Why would a Ti-alloy taper pair be used when this is not relevant to total hip replacements? Suggest using more descriptive and intuitive labels.<br>Thank you very much for this important note. We see that the table was quite confusing and therefore we simplified the table and described the groups in the text more precisely.<br><br>Results<br>Lines 192 and 199: I don't know what mismatch means in this context since it is listed for the inner tapers or outer tapers. Difference from the intended value of 5.665? Mismatch usually refers to the difference between inner and outer tapers.<br>Thank you very much for this important comment. We changed the wording in the section to taper "deviation from nominal value" instead of mismatch (L206-214). And we have added an additional table with the mismatch values to the supplement<br><br>The combinations of pairs is very confusing, similar to my comment in the methods. Suggest adding a table that explicitly states which inner taper is paired with which outer taper and the resulting angular mismatch.

Powered by Editorial Manager® and ProduXion Manager® from Aries Systems Corporation

Thank you very much for your comment. As written above we simplified the first table and additionally we added another table in the supplemental material with the angular mismatch of every taper pair.

Figure 3: Please use a more descriptive caption. I assume these are all inner tapers because of the process parameters listed.
Thank you very much for the note. We added the missing information in the figure caption.
Even with turning, these would still be considered very smooth inner tapers. Most stem tapers that are turned have trough to peak heights from 10 to 13 microns instead of 3 microns. This is fine, but worthy of a discussion point.
In the basic research oriented approach of this study on the effects of adhesive bonding we chose to investigate the smooth vs. rough surfaces with of a roughness of Rz = 5 µm and Rz = 15 µm respectively. This is admittedly somewhat different than current implants. We have added a sentence to the discussion in this regard (L302-304).

Line 242: Does the free volume take into account all surface differences (angular mismatch, sandblasting, turn machining parameter differences that results in greatly different surface contacts)? It does not seem surprising that there is no correlation between free volume and push-out.
The calculated free volume actually includes the taper mismatch and the roughness profile resulting from the machining process of the trunnion and the taper. We have added a sentence to the discussion in this regard (L250-252).
Lines 256-261: not sure what the purpose of this paragraph is, steeper/not steeper is relevant to a stronger taper pair?
Reviewer#1 raised the question of what influence the stiffness of the test setup has on the results in Figure 6. For this reason, we determined the stiffness of 39,111N/mm for the setup and corrected the data for this.
The gradient of the force-path curves already say something about the stability of the cone connection. the pairings with a greater gradient also tend to have higher force values. And there is less movement in the connection under load.

Discussion
Lines 272-274: what data is this discussion point referring to? It needs to be in the introduction if important.
Thank you very much for this important question. We elaborated this section with more details to make it more understandable (L281-288).

Lines 278-281: Need to discuss the angular mismatch in relation to what is normal for actual THA. Many of the ones in this study would be outliers and not used in clinical practice therefore the conclusions drawn are not relevant/helpful
Thank you for your comment, we have addressed the issue of describing taper-mismatch more clearly in response to a comment from the first reviewer. We now report the taper mismatch of the specimens tested (L211-214 and table in supplemental material), and that these did not significantly affect push out-force in the passage of the discussion the reviewer was referring to (L281-288).

Lines 306-308: What does suitable mean in this context? What about the other studies in the literature? There have been EXTENSIVE studies with pull out/push out etc. The conversation about "suitable" is irrelevant to the paper. Recommend only saying that sandblasted surfaces resulted in the highest push-out forces that were greater than 2 kN.
Thank you for insight, you are of course correct. We have reformulated the paragraph and added the relevant literature. Our goal was however not to show that adhesively bonded tapers are better than any taper ever tested, but that in the system we investigated bonding results in significant increases of push-out force.

The limitations section is not extensive enough. (lines 329-330). What are the more realistic conditions? What about realistic taper mismatch, etc?
Thank you very much for this valuable comment. We extended the limitation section with further points and information (L344-350). Please also see comment on L278-281.

Conclusion

	Line 334-335: Please remove "generally accepted". There is no generally accepted value in this field.<br>Thank you very much for this observation. We have revised the sentence as follows: “The push-out forces generated from the sandblasted tapers resulted in the highest push-out forces and were greater than push out forces from mechanical assembled taper pairs reported in the literature.”<br><br>All figures and tables: use better abbreviations or spell out entire words. Figures and tables are difficult to understand<br>Thank you very much for this important note. We changed the figure captions of figure 3,5 and 6 as well as the tables 1 and 2 to make them more understandable.
Additional Information:	
Question	Response
**Financial Disclosure**<br><br>Enter a financial disclosure statement that describes the sources of funding for the work included in this submission. Review the submission guidelines for detailed requirements. View published research articles from *PLOS ONE* for specific examples.<br><br>This statement is required for submission and **will appear in the published article** if the submission is accepted. Please make sure it is accurate.<br><br>**Funded studies**<br>Enter a statement with the following details:<br>• Initials of the authors who received each award<br>• Grant numbers awarded to each author<br>• The full name of each funder<br>• URL of each funder website<br>• Did the sponsors or funders play any role in the study design, data collection and analysis, decision to publish, or preparation of the manuscript?<br><br>Did you receive funding for this work?	Yes
Please add funding details.<br>as follow-up to "**Financial Disclosure**<br><br>Enter a financial disclosure statement that describes the sources of funding for the work included in this submission. Review the submission guidelines for detailed requirements. View published research articles from *PLOS ONE* for specific	Deutsche Forschungsgemeinschaft (DFG, German Research Foundation) within the SFB/TRR-298-SIIRI-Project-ID 426335750 “Safety integrated and infection reactive implants”.<br>Publication costs are covered by the German Research Foundation (DFG) and the Open Access Publication Fund of Hannover Medical School (MHH).

examples.<br><br>This statement is required for submission and **will appear in the published article** if the submission is accepted. Please make sure it is accurate.<br><br>**Funded studies**<br>Enter a statement with the following details:<br>• Initials of the authors who received each award<br>• Grant numbers awarded to each author<br>• The full name of each funder<br>• URL of each funder website<br>• Did the sponsors or funders play any role in the study design, data collection and analysis, decision to publish, or preparation of the manuscript?<br><br>Did you receive funding for this work?"	
Please select the country of your main research funder (please select carefully as in some cases this is used in fee calculation).<br>as follow-up to "**Financial Disclosure**<br><br>Enter a financial disclosure statement that describes the sources of funding for the work included in this submission. Review the submission guidelines for detailed requirements. View published research articles from *PLOS ONE* for specific examples.<br><br>This statement is required for submission and **will appear in the published article** if the submission is accepted. Please make sure it is accurate.	GERMANY - DE

**Funded studies**<br>Enter a statement with the following details:<br>• Initials of the authors who received each award<br>• Grant numbers awarded to each author<br>• The full name of each funder<br>• URL of each funder website<br>• Did the sponsors or funders play any role in the study design, data collection and analysis, decision to publish, or preparation of the manuscript?<br><br>Did you receive funding for this work?"	
**Competing Interests**<br><br>Use the instructions below to enter a competing interest statement for this submission. On behalf of all authors, disclose any competing interests that could be perceived to bias this work—acknowledging all financial support and any other relevant financial or non-financial competing interests.<br><br>This statement is required for submission and **will appear in the published article** if the submission is accepted. Please make sure it is accurate and that any funding sources listed in your Funding Information later in the submission form are also declared in your Financial Disclosure statement.<br><br>View published research articles from *PLOS ONE* for specific examples.	The authors have declared that no competing interests exist.

**NO authors have competing interests**<br><br>Enter: *The authors have declared that no competing interests exist.*<br><br>**Authors with competing interests**<br><br>Enter competing interest details beginning with this statement:<br><br>*I have read the journal's policy and the authors of this manuscript have the following competing interests: [insert competing interests here]*<br><br>* typeset	
**Ethics Statement**<br><br>Enter an ethics statement for this submission. This statement is required if the study involved:<br><br>• Human participants<br>• Human specimens or tissue<br>• Vertebrate animals or cephalopods<br>• Vertebrate embryos or tissues<br>• Field research<br><br>Write "N/A" if the submission does not require an ethics statement.<br><br>General guidance is provided below. Consult the submission guidelines for detailed instructions. **Make sure that all information entered here is included in the Methods section of the manuscript.**	N/A

Powered by Editorial Manager® and ProduXion Manager® from Aries Systems Corporation

**Format for specific study types**<br><br>**Human Subject Research (involving human participants and/or tissue)**<br>• Give the name of the institutional review board or ethics committee that approved the study<br>• Include the approval number and/or a statement indicating approval of this research<br>• Indicate the form of consent obtained (written/oral) or the reason that consent was not obtained (e.g. the data were analyzed anonymously)<br><br>**Animal Research (involving vertebrate animals, embryos or tissues)**<br>• Provide the name of the Institutional Animal Care and Use Committee (IACUC) or other relevant ethics board that reviewed the study protocol, and indicate whether they approved this research or granted a formal waiver of ethical approval<br>• Include an approval number if one was obtained<br>• If the study involved *non-human primates*, add *additional details* about animal welfare and steps taken to ameliorate suffering<br>• If anesthesia, euthanasia, or any kind of animal sacrifice is part of the study, include briefly which substances and/or methods were applied<br><br>**Field Research**<br><br>Include the following details if this study involves the collection of plant, animal, or other materials from a natural setting:<br>• Field permit number<br>• Name of the institution or relevant body that granted permission	
**Data Availability**<br><br>Authors are required to make all data underlying the findings described fully available, without restriction, and from the time of publication. PLOS allows rare exceptions to address legal and ethical concerns. See the PLOS Data Policy and FAQ for detailed information.	Yes - all data are fully available without restriction

A Data Availability Statement describing where the data can be found is required at submission. Your answers to this question constitute the Data Availability Statement and **will be published in the article**, if accepted.<br><br>**Important:** Stating 'data available on request from the author' is not sufficient. If your data are only available upon request, select 'No' for the first question and explain your exceptional situation in the text box.<br><br>Do the authors confirm that all data underlying the findings described in their manuscript are fully available without restriction?	
**Describe where the data may be found in full sentences. If you are copying our sample text, replace any instances of XXX with the appropriate details.**<br><br>• If the data are **held or will be held in a public repository**, include URLs, accession numbers or DOIs. If this information will only be available after acceptance, indicate this by ticking the box below. For example: *All XXX files are available from the XXX database (accession number(s) XXX, XXX.*).<br>• If the data are all contained **within the manuscript and/or Supporting Information files**, enter the following: *All relevant data are within the manuscript and its Supporting Information files.*<br>• If neither of these applies but you are able to provide **details of access elsewhere**, with or without limitations, please do so. For example:<br><br>*Data cannot be shared publicly because of [XXX]. Data are available from the XXX Institutional Data Access / Ethics Committee (contact via XXX) for researchers who meet the criteria for access to confidential data.*<br><br>*The data underlying the results presented in the study are available from (include the name of the third party*	All relevant data are within the manuscript and its Supporting Information files.

and contact information or URL).<br>• This text is appropriate if the data are owned by a third party and authors do not have permission to share the data.<br><br>* typeset	
Additional data availability information:	

Manuscript

Click here to access/download;Manuscript;Manuscript without track changes.docx

Influence of the use of an adhesive connection on the joint strength of modular hip endoprostheses

Ann-Kathrin Einfeldt[a], Beate Legutko[b], Philipp-Cornelius Pott[c], Benjamin Bergmann[b], Berend Denkena[b], Christof Hurschler[a], Bastian Welke[a]*

[a] Laboratory for Biomechanics and Biomaterials, Department of Orthopedic Surgery – Annastift DIAKOVERE, Hannover Medical School, Anna-von-Borries-Str. 1-7, 30625 Hannover, Germany

[b] Institute of Production Engineering and Machine Tools, Leibniz University Hannover, An der Universität 2, 30823 Garbsen, Germany

[c] Department of Prosthetic Dentistry and Biomedical Materials Research, Hannover Medical School, Carl-Neuberg Str. 1, 30625 Hannover, Germany

*Corresponding author:

Bastian Welke (BW)

Welke.bastian@mh-hannover.de

ORCID ID: 0000-0003-2526-8129

Abstract

Introduction

Modular hip implants enables a more precise adaptation of the prosthesis to the patient's anatomy. However, they also carry the risk of increased revision rates due to micromotion at the taper junction. In order to minimize this risk, one potential solution is to establish an adhesive bond between the metal taper junctions. Load-stable bonding techniques, already successfully employed in dentistry for connecting materials such as metals and ceramics or different alloys, offer a promising approach. Nevertheless, the bond strength of tapered adhesive bonds in modular hip implants has not been investigated to date.

Materials and Methods

Twenty-eight tapered junctions, consisting of a taper (female taper) and a trunnion (male taper) were turned using TiAl6V4 ELI (n = 16) and CoCr28Mo6 (n = 12). The process parameters cutting speed (v_c = 50 m/min or 100 m/min) and feed (f = 0.1 mm, 0.05 mm or 0.2 mm) were varied for the trunnions. For each set of process parameters, one trunnion and one taper were additionally subjected to sandblasting. To investigate the effect of geometry, angular mismatch in the samples were measured. The taper pairs were bonded with a biocompatible adhesive, and push-out tests were subsequently performed.

Results

The push-out forces generated from the taper connections where both tapers were sandblasted showed a mean push-out force of 5.70 kN. For the samples with only the trunnion sandblasted, the mean force was 0.58 kN, while for the samples with only taper sandblasted the mean push-out force was 1.32 kN. When neither of the tapers was sandblasted the mean push-out force was 0.91 kN. No significant effect of the process parameters on the push-out force was observed. Only the reduced valley depth Svk showed a slight correlation for the CoCr28Mo6

samples ($R^2 = 0.54$). The taper pairs with taper mismatch (between trunnion and taper) greater than |0.1°| did not show lower push-out forces than the specimens with lower taper mismatch.

Conclusions

Sandblasted and adhesive-bonded tapered connections represent a viable suitable alternative for modular hip implant connections. Slight differences in taper geometry do not result in reduced push-out forces and are compensated by the adhesive. In mechanically joined tapers these differences can lead to higher wear rates. Further investigation under realistic test conditions is necessary to assess long-term suitability.

Keywords: modular hip endoprostheses, adhesive connection, junction strength, taper junction

Introduction

Total hip arthroplasty (THA) is one of the most common inpatient surgeries, with approximately 240,000 procedures performed each year [1]. Since the late 1990s, modular hip endoprostheses have allowed surgeons to better address individual patient characteristics by using specific implant components with different stem neck sizes and angles (1, 2)]. For example, bi-modular tapered neck adapters allowed the generation of different antetorsion and caput-collum-diaphyseal (CCD) angles, facilitating optimal reconstruction of the biomechanics of the joint (1)].

Nonetheless, the advantages of bi-modularity were associated with increased complications, resulting in revision rates of 6% compared to 3% for monolithic prosthesis systems after 5 years (3)]. Typical damage observed is the clinical failure of tapered stem junctions due to micromotion, which appear to be dependent on the design and material coupling (Co-Cr29-Mo vs. Ti-6Al-4V) at the junction between the stem and the adapter (2, 4)]. For instance, titanium neck adapters exhibited significantly larger micromotions than cobalt-chromium adapters and

contaminated interfaces also showed significantly larger micromotions (4)]. These micromotions can lead to fretting and crevice corrosion at the taper junction, potentially causing premature connection failure accounting for 2% to 3% of all THA revisions (5, 1)].

Despite being a well-known phenomenon the exact causes of theses complications remain poorly understood. However, a number of mechanisms and possible causes are commonly cited, such as galvanic and crevice corrosion, micromotions and fretting, and contamination of the joint leading to the release of metal ions (6)]. Furthermore, modularity plays a central role in the damage tolerance of implants, and each additional modular connection therefore increases the effect.

Another aspect leading to higher wear rates during dynamic loading is a so-called taper mismatch (7, 8)]. It describes the difference of the angle between the trunnion and the taper of the hip implant and is sometimes designed into the systems to ensure consistent performance (8)]. Nevertheless, a few finite element studies found out that smaller mismatches result into less wear in modular hip implants (7, 8)]. Taper mismatch can be negative (larger taper than trunnion), or positive (smaller taper than trunnion). Ahkanfar et al. performed a comparative FEM study of the effect of different taper mismatch angels and suggested that taper mismatch should be manufactured with to less than 6’ or 0,1° (9)].

The above mentioned possible causes of taper junction failure area multiple and have diverse causes. Despite their utility in restoring patient specific geometries, bi-modular hip implants have widely been recalled from the market due to the relatively high rates of failure observed. One possible method to address the aforementioned failure mechanisms (micromotion, fretting-corrosion, contamination, and taper-missmatch tolerances) may be to establish an adhesive bond in the metal taper junctions. Such an adhesive bond has the potential to enhance the mechanical load transfer between the metal taper partners and to prevent corrosion and release of particles and ions into the joint. These types of adhesively joined interfaces are not new. In

dentistry, adhesive techniques have been well established for bonding various materials in different types of dentures and crowns (10–13)]. Thus, titanium and cobalt-chromium tapered bonds have successfully been utilized and investigated in this context (14, 15)]. Furthermore, several bonding systems have been reported and the use of adhesives proven to be effective for bonding titanium alloys (15)]. Bonding systems investigated in this context have included different primers and luting agents, often in conjunction with surface modification such as airborne-particle abrasion, a specialized surface treatment technique similar to sandblasting (16)]. The dental application most comparable to the taper-junctions addressed in this study are hybrid abutments, where the respective restoration is bonded to standardized implant abutments (17)]. However, the bond strength of tapered adhesive bonds as used in in modular hip implants has not been investigated to date. Due to the high revision rates in bi-modular hip implants, they widely disappeared from the market. Nevertheless, this study aims to investigate adhesive bonding with the aim of addressing the issues that led to their failure. The aim of this study was therefore to perform an exploratory investigate of the possible suitability of using an adhesive bond to join modular hip implant junctions. To achieve this goal, various process parameters and the resulting surface topographies of titanium and cobalt chromium bonded joints were examined in relation to their bond strength.

Materials and Methods

Specimen preparation

In total 28 taper connections consisting of a trunnion (male taper) and a taper (female taper) were manufactured. The tapers were manufactured on a linear lathe (CTX 420, Gildemeister, Wernau, Germany) and the trunnions on the turnmill center (NTX 1000, DMG MORI, Wernau, Germany). For turning the trunnion, indexable inserts (type VBMT 1604 MM 1115, Sandvik Tooling Deutschland GmbH, Duesseldorf, Germany) were used. The trunnion has a nominal taper angle of θ_{AK} = 11.33°, as well as the diameters of d_E = 12.74 mm and d_H = 14.88 mm. The

length of the angular surface in relation to the central axis of the trunnion was l_{AK} = 11.22 mm and the length of the bore of the taper was l_{IK} = 14 mm to ensure that in case of minor deviations of the geometry due to the manufacturing process, the entire taper length is still in contact at the junction.

The process parameters cutting speed (v_c = 50 m/min or 100 m/min) and feed (f = 0.1 mm, 0.05 mm or 0.2 mm) were varied for the trunnions, resulting in a total of seven different testing groups, each consisting out of four taper pairs (table 1). In the first four groups the taper pairs (Ti 1 – Ti 4) were made out of TiAl6V4 ELI whereas in the remaining three groups the samples were manufactured out of CoCr28Mo6 (CoCr 5-CoCr 7). The depth of cut a_p = 0.25 mm were kept constant. The process parameters for the tapers were not varied to ensure that all observed effects were dependent on the surface topography of the trunnions. The tapers were manufactured with indexable inserts (type CCMT 060204 Sandvik Tooling Deutschland GmbH, Duesseldorf, Germany) on a boring bar. The machining process was performed with v_c = 70 m/min, f = 0.1 mm and ap = 0.25 mm. In each testing group one trunnion and one taper were additionally sandblasted (sb). In groups Ti 2, Ti 4, CoCr 5 and CoCr 7 both tapers of a taper pair were sandblasted whereas in the remaining three groups different tapers were sandblasted (table in supplemental material). The sandblasting pressure was set to p = 2.3 bar, and the taper contact surface was manually blasted with a grain size of x = 101 µm corundum at approximately 10 mm distance from the surface for 30 seconds.

Table 1: Overview over the different process parameters for each taper group. Tapers in group 1-4 were manufactured from TiAl6V4 ELI, tapers in group 5-7 were manufactured from CoCr28Mo6 (shaded in grey).

	**v_c [m/min]**	**f [mm]**
Ti 1	50	0.10

Ti 2	100	0.05
Ti 3	50	0.05
Ti 4	100	0.20
CoCr 5	50	0.10
CoCr 6	100	0.05
CoCr 7	50	0.05

Measurement of the samples

As possible angular mismatch of the tapers can also influence the separating forces that occur in the taper connections, a 3D coordinate measurement system (Reference Xi 1076, Leitz, Stockholm, Sweden) was used to determine the resulting geometry of the manufactured tapers.

Subsequently, roughness measurements were performed for the trunnion and tapers. The tapers were scanned over a distance of 8 mm (MarSurf LD 130 with the LP C 3.5-102/90° probe, Mahr, Göttingen, Germany). The probe then repeated this process a further nine times at different starting points in order to obtain a more accurate representation of the surface topography across a wider measuring distance.

The roughness measurement of the trunnions was conducted using an optical measuring device over a length of 5 mm (Duo Vario, Confovis, Neu-Isenburg, Germany). Measurements were taken for all tapers, starting from the main diameter in the direction of the end diameter and from the end diameter in the direction of the main diameter. This approach resulted in a total measuring length of 10 mm per taper, allowing for the analysis of potential deviations in the surface over the entire contact area of the taper.

The measurement data was analysed using the MountainsMap analysis programme (Digital Surf, 8.2, Besançon, France). The characteristic values of the surface topographies were determined. The roughness parameters maximum height of profile (Rz) and maximum depth of

profile (Rv) according to DIN EN ISO 21920 and the primary surface topography profile (Reduced Peak Hight (Spk), Reduced Valley Depth (Svk)) according to DIN EN ISO 25178, were recorded over a length ranging from 3.8 to 4.6 mm.

Bonding the tapers

After manufacturing and blasting, the tapers were bond with a biocompatible adhesive system. The adhesive is a self-curing luting composite used for the adhesive luting of metal and metal-ceramics, among other materials. The adhesive system used (Multilink Automix, Ivoclar Vivadent AG, Ellwangen, Germany) is a self-curing material consisting of a 2-component primer system (Multilink Primer A + B) and a 2-component luting resin. Before bonding, the tapers were cleaned in an ultrasonic bath with alcohol to eliminate any negative influences of possible contamination on the bond.

The two primer components A and B were mixed in a 1:1 ratio and evenly applied to the contact surfaces without the formation of drops until the surface was completely wetted. The exposure time for the primer was 2 minutes. The surfaces that needed to be free of the luting resin were isolated with cling film. The luting resin was then applied bubble-free into the opening of the taper using the application cannula and the tapers were joined together (Figure 2). The trunnion was inserted into the taper and allowed to sag under its own weight until the adhesive oozes out at the edge of the contact surface. This excess adhesive can be removed within the curing time of 8 to 10 minutes at 23°C ± 1°C (Figure 2).

Figure 2: Schematic illustration of the individual processing steps and the setup for the push-out tests

Push-out tests

The push-out tests were performed using a universal testing machine (8501, Instron, Illinois Tool Works Inc., Norwood, USA). The tests were carried out after a curing time of 24 hours at 23°C ± 1°C of the probes. The machine's stamp moved down hydraulically at a velocity of 0.48 mm/min, pressing the trunnion out of the taper. At every 0.001 mm displacement of the hydraulic cylinder, the following parameters were recorded: time, axial force and axial displacement. The measured displacement of the testing machine was subsequently corrected for the rigidity of the setup.

Results

Taper geometry

The main diameter of the trunnion was targeted to be $d_M = 14.88$ mm, the end diameter $d_E = 12.74$ mm, the taper length $l = 11.22$ mm, and the angle $\theta_n = 11.33°$.

All trunnions show deviations from the nominal manufacturing values, with a mean deviation of all trunnions was 0.18 ± 0.19 mm, whereas the Ti 3-row showed the smallest deviations (Deviation $d_M = 0.06$ mm). The TiAl6V4 ELI tapers tend to be above, and the CoCr28Mo6 tapers below the nominal values. The mean deviation of the end diameter for all trunnions was 0.22 ± 0.23 mm, and for the taper length 0.03 ± 0.03 mm. The Ti 2-row shows the smallest deviations from the nominal value for both parameters (Deviation $d_E = 0.04$ mm, Deviation $l = 0.004$ mm). CoCr28Mo6 tapers showed greater dispersions than the TiAl6V4 ELI tapers. The mean angle deviation of all trunnions is 0.04 ± 0.02°, with the Ti 3-row again showing the smallest deviations ($\theta = 0.02°$) from the nominal manufacturing value whereby and the CoCr28Mo6 tapers exhibiting greater deviations than the TiAl6V4 ELI tapers.

The face diameter of the taper was aimed to be $d_F = 14.88$ mm, the end diameter $d_E = 12.74$ mm and the angle $\theta_n = 11.33°$.

All face diameters of the tapers are above the nominal value (Deviation $d_F = 0.11 \pm 0.02$ mm) whereas in the end diameter they are below the nominal value (Deviation $d_E = 0.37 \pm 0.02$ mm). The mean deviation of the nominal angle of all tapers is 0.64 ± 0.02° and is above the nominal value for all tapers.

Due to the deviations from the nominal values different free volumes - areas where there is no contact between the tapers - exist for the taper pairs. All 28 taper pairs exhibit a negative taper mismatch (trunnion angle < taper angle) and therefore a proximal contact. All taper pairs show a taper mismatch greater than |0.1°| with a mean of -0.182 ± 0.047° (range -0.012° to -0.369°) (table in supplemental material).

Roughness

The roughness measurement of the trunnions after sandblasting shows the greatest roughness in the Ti 4-group, followed by the CoCr 5 and Ti 1 groups (Table 2). CoCr28Mo6 tapers exhibit greater Rz and Rv values than TiAl6V4 ELI tapers when the same process parameters are used. Ti 4 group reaches the greatest Spk, followed by the Ti 1 and the CoCr 5 group. CoCr 5 group show the greatest Svk.

Table 2: Overview over the different roughness and process parameters for each sample group of trunnions before (shaded in blue) and the one trunnion per sample group after sandblasting; Legend: Rz = maximum height of profile; Rv = maximum depth of profile; Spk = Reduced Peak Hight; Svk = Reduced Valley Depth

	**Mean Rz [µm]**	**Mean Rv [µm]**	**Mean Spk [µm]**	**Mean Svk [µm]**	**Rz [µm]**	**Rv [µm]**	**Spk [µm]**	**Svk [µm]**
Ti 1	4.812	2.335	1.218	0.685	4.531	2.014	1.437	0.932
Ti 2	1.892	0.968	0.330	0.396	2.027	1.118	1.054	0.983
Ti 3	2.412	1.221	0.615	0.394	1.495	0.817	1.004	1.085

Ti 4	15.080	5.470	8.554	2.326	12.500	4.705	6.625	1.115
CoCr 5	5.057	2.673	0.837	1.316	6.595	4.164	0.798	3.035
CoCr 6	2.606	1.274	0.475	0.375	2.175	1.090	1.010	0.744
CoCr 7	2.854	1.508	0.480	0.689	2.488	1.515	0.813	1.331

After sandblasting, the trunnions show a more porous surface. For the TiAl6V4 ELI tapers the Rz and Rv values hardly change whereas for the CoCr28Mo6 tapers these values were reduced after sandblasting. Both the Spk and the Svk increases after sandblasting for both materials.

As there was no variation in the process parameters for the tapers, only differences between the sandblasted and not sandblasted tapers were evaluated. The sandblasted surface is more porous (Figure 3, Supplement) leading to increased roughness parameters compared to the untreated surface.

Figure 3: Surface and profile plots of a turned sample (left in each case) and a turned and subsequently sandblasted sample (right in each case) of a trunnion.

<u>Push-out tests</u>

The results of the push-out tests show the greatest forces ($mean_F$ = 5.70 kN) for the taper connections where both tapers were sandblasted (Figure 4, Supplement). All other pairing situations show smaller forces (only taper sandblasted $mean_F$ = 1.32 kN, no sandblasting $mean_F$ = 0.91 kN), with the smallest force when only the trunnion was sandblasted (meanF = 0.58 kN).

Figure 4: Results of the push-out test for all taper connections and materials divided into groups by the sandblasting (sb) situation. Values above and below 1.5 times the interquartile range are shown as outliers.

To investigate whether the mismatches in taper geometry influence the resulting push-out forces the free volume for each taper pair was calculated. The calculated free volume includes the taper mismatch and the roughness profile resulting from the machining process of the trunnion and the taper. No correlations between free volume and therefore the adhesive volume and the push-out forces could be seen.

When looking at the push-out forces in relation to the different process parameters and therefore the surface topographies, no correlation between the roughness parameters Rz and Rv and the push-out force were observed ($R^2_{Rz} = 0.03$; $R^2_{Rv} = 0.08$).

No correlations were observed ($R^2_{allsamples} = 0.02$, $R^2_{Ti}= 0.02$, $R^2_{CoCr} = 0.13$) for the push-out forces in relation to the tip height. For the Svk values, a slight correlation ($R^2_{CoCr} = 0.54$, $p = 0.08$) can be observed especially for the CoCr28Mo6 samples (Figure 5, Supplement).

Figure 5: Correlation between Svk (Reduced Valley Depth) and push-out force for all TiAl6V4 ELI samples (blue) and CoCr28Mo6 samples (grey). Dotted line shows the resulting regression line for each material type.

The force-distance curves of the push-out tests are exemplarily shown for individual samples of each group (see Fig 6). The graph shows the smallest incline for the only turned CoCr28Mo6 sample (Fig. 6, red line). For the only turned TiAl6V4 ELI sample the incline is steeper (Fig. 6, yellow line). When both tapers of one sample are sandblasted the steepest inclination profiles

are seen, (Fig. 6, light and dark blue). When one sample is sandblasted and one sample is turned, no clear inclination profile can be observed for the samples.

Figure 6: Force-Path curves of the push-out tests exemplarily shown for individual samples of the different groups (CoCr28Mo6 only turned = red; TiAl6V4 ELI only turned = yellow; CoCr28Mo6 both sandblasted = light blue; TiAl6V4 ELI both sandblasted = dark blue. The measured displacement of the testing machine was corrected for the stiffness of the setup.

A visual analysis of the tapers after the push-out tests showed, that the adhesive adheres only to the sandblasted surfaces. No adhesive residues were observed macroscopically at the surfaces that were only turned.

Discussion

Taper geometry

The smallest deviations in taper geometry could be observed when the cutting speed and the feed were both small as shown in the Ti_3 group with a d_M = 0.06 mm (v_c = 50 m/min and f = 0.05 mm). The feed seems to have a greater influence on the precision of the taper geometry than the speed, as both groups with a feed of 0.05 mm (Ti_2 and Ti_3) show the smallest deviations from nominal values even though different speeds were used. CoCr28Mo6 trunnions tend to be below the nominal values whereas TiAl6V4 tend to be above them. The tapers show that the face diameter of all tapers lies above whereas the end diameter lies below the nominal value. This is due to the different tool deflection caused by the different material properties.

When comparing the taper material, CoCr28Mo6 shows greater deviations from the nominal values than TiAl6V4 ELI. As CoCr28Mo6 is harder than TiAl6V4 ELI, this could be the reason. Overall, the results show that the material has a greater influence on the deviations on taper geometry than the process parameters.

All 28 samples show a proximal contact area. Bitter et al. (8) recommend that a distal contact area should be chosen to reduce the wear rate. As there only samples showing a proximal contact area no conclusion can be drawn about which contact area is to be preferred in adhesive bonded taper pairs.

All taper pairs show a taper mismatch greater than |0.1°|. The results of the push-out tests indicate that there is no correlation between the amount of mismatch and the push-out force. Therefore, the adhesive appears to compensate slight differences in taper geometry and consequently taper mismatch.

Roughness

Most stem tapers that are turned have trough to peak heights form 10 to 13 µm. In order to investigate the influence of the surface topographie on the push-out force the range of the surface roughness is from Rz = 5 µm to Rz = 15 µm. CoCr28Mo6 was observed to have greater Rz and Rv roughness than TiAl6V6 ELI. Reduced peak height on the other hand was greater for Ti than for CoCr28Mo6. Before sandblasting, it could be shown that the feed has the greatest influence on the roughness, whereas the speed does not seem to influence this parameter.

After sandblasting, the roughness parameters hardly change for the TiAl6V6 ELI trunnions whereas for the CoCr28Mo6 tapers roughness is reduced. It cannot be ruled out that this difference is due to the manual sandblasting, which might have led to inconsistent influences on the surface. When looking at the surface topographies before and after sandblasting it is clear that the surface topography after sandblasting is more stochastic and the possible bonding surface is enlarged. For tapers the roughness increases after sandblasting regardless of the material.

Push-out tests

A preliminary study on taper junctions of the same geometry and materials showed that tapers with an assembled at a speed of v = 8 µm/s and a force of up to F = 4kN reached push-out forces between $F_{separate}$ = 0.79 and 1.8 kN (18). The push-out tests in this study show that taper connections where both tapers were sandblasted before bonding have the greatest push-out forces, ranging from 4.4 to 7.1 kN. These adhesively bonded junctions thus exceeded native taper junctions by a minimum of 244 %. Other studies of native taper pull out force have reported ranges of pull-off force under normal conditions for different material pairings and surface modifications ranging from 1.6kN to 3kN (19–21), a study using very high impaction forces of 14kN reported pull-off forces of up to 6kN (22). Mechanically, the push-out test we performed is equivalent to the pull off test as described in the cited studies.

The Svk shows a slight correlation with the resulting push-out force especially for the CoCr28Mo6 tapers whereas all other roughness parameters are not showing any correlations. The greater the Svk the more liquid and in this case, adhesive, can be collected on the surface of a sample. A greater amount of adhesive on the surface therefore leads to increased bonding strength. After sandblasting this parameter increases for all the samples. This is in accordance with the increased push-out forces for the sandblasted taper connections. Also, the force-displacement curves and the visual analysis of the tapers support this thesis. The steeper inclination of the force time curve for the sandblasted samples, indicate a stiffer junction of the taper pairs as the adhesive adheres to both taper partners. Sandblasting leads to a more porous surface where the adhesive is more likely to adhere to than to only turned samples. This could be seen in the visual analysis after the push-out test.

Even though the other roughness parameters did not influence the push-out forces, connections where only the taper was sandblasted show higher push-out forces than taper connections without sandblasting or with only trunnion blasting. As the sandblasted tapers exhibit increased roughness parameters, a slight influence of the roughness of sandblasted tapers can be assumed.

Nevertheless, the greatest bonding strength is achieved when both tapers are sandblasted, which is why further investigations should be conducted in this manner. In further investigations, adhesive bonded taper connections should be tested under more realistic conditions. Micromotion is the most common cause of failure of modular implant connections. These possible micromotions were not investigated in our study. This is the subject of further planned fatigue tests with 10 million load cycles under standardised testing conditions.

In the current study, we investigated taper combinations consisting of both partners being either TiAl6V6 ELI or CoCr28Mo6. Future investigations should also test the combination of these two materials. Furthermore, further research will investigate possible methods of revising of glued taper junctions.

Conclusion

The push-out forces generated from the sandblasted tapers resulted in the highest push-out forces and were greater than push-out forces from mechanical assembled taper pairs reported in the literature. Therefore, the conclusion can be drawn that sandblasted and adhesive bonded taper connections are promising alternative for modular hip implant junctions. Slight differences in taper geometry are compensated by the adhesive and do not influence the push-out forces. Further investigation under realistic testing conditions is necessary to assess the suitability in the long-term.

List of abbreviations

CCD:	caput-collum-diaphyseal
f:	feed
Rv:	maximum depth of profice

Rz:	maximum height of profile
Sb:	sandblasted
Spk:	reduced peak hight
Svk:	reduced valley depth
THA:	Total hip arthroplasty
v_c:	cutting speed

References

1. Grupp TM, Weik T, Bloemer W, Knaebel H-P. Modular titanium alloy neck adapter failures in hip replacement--failure mode analysis and influence of implant material. BMC Musculoskelet Disord 2010; 11:3.

2. Jauch SY, Huber G, Haschke H, Sellenschloh K, Morlock MM. Design parameters and the material coupling are decisive for the micromotion magnitude at the stem-neck interface of bi-modular hip implants. Med Eng Phys 2014; 36(3):300–7.

3. National Joint Replacement Registry, Australian Orthopaedic Association. 2018 Hip, Knee & Shoulder Arthroplasty Annual Report; 2018.

4. Jauch SY, Huber G, Hoenig E, Baxmann M, Grupp TM, Morlock MM. Influence of material coupling and assembly condition on the magnitude of micromotion at the stem-neck interface of a modular hip endoprosthesis. J Biomech 2011; 44(9):1747–51.

5. Jauch SY, Huber G, Sellenschloh K, Haschke H, Baxmann M, Grupp TM et al. Micromotions at the taper interface between stem and neck adapter of a bimodular hip prosthesis during activities of daily living. J Orthop Res 2013; 31(8):1165–71.

6. Gilbert JL, Buckley CA, Jacobs JJ. In vivo corrosion of modular hip prosthesis components in mixed and similar metal combinations. The effect of crevice, stress, motion, and alloy coupling. J Biomed Mater Res 1993; 27(12):1533–44.

7. Gustafson JA, Mell SP, Levine BR, Pourzal R, Lundberg HJ. Interaction of surface topography and taper mismatch on head-stem modular junction contact mechanics during assembly in modern total hip replacement. J Orthop Res 2023; 41(2):418–25. Available from: URL: https://pubmed.ncbi.nlm.nih.gov/35488727/.

8. Bitter T, Khan I, Marriott T, Lovelady E, Verdonschot N, Janssen D. The effects of manufacturing tolerances and assembly force on the volumetric wear at the taper junction in modular total hip arthroplasty. Comput Methods Biomech Biomed Engin 2019; 22(13):1061–72.

9. Ashkanfar A, Langton DJ, Joyce TJ. A large taper mismatch is one of the key factors behind high wear rates and failure at the taper junction of total hip replacements: A finite element wear analysis. Journal of the mechanical behavior of biomedical materials 2017; 69:257–66. Available from: URL: https://pubmed.ncbi.nlm.nih.gov/28110182/.

10. Khasawneh S, al-Wahadni A, Lloyd CH. Comparison of bond strengths between adhesive and conventional acrylic resins to cobalt chromium denture base alloy. Eur J Prosthodont Restor Dent 2003; 11(3):119–24.

11. Pott PC, Stiesch M, Eisenburger M. Influence of artificial aging on the shear bond strength of zirconia-composite interfaces after pretreatment with new 10-MDP adhesive systems. Journal of Dental Materials and Techniques 2016; 5(1).

12. Pott P-C, Syväri T-S, Stiesch M, Eisenburger M. Influence of nonthermal argon plasma on the shear bond strength between zirconia and different adhesives and luting composites after artificial aging. J Adv Prosthodont 2018; 10(4):308–14.

13. Kim D-S, Ahn J-J, Kim G-C, Jeong C-M, Huh J-B, Lee S-H. Influence of Non-Thermal Atmospheric Pressure Plasma Treatment on Retentive Strength between Zirconia Crown and Titanium Implant Abutment. Materials (Basel) 2021; 14(9).

14. Taira Y, Yanagida H, Matsumura H, Yoshida K, Atsuta M, Suzuki S. Adhesive bonding of titanium with a thione-phosphate dual functional primer and self-curing luting agents. Eur J Oral Sci 2000; 108(5):456–60.

15. Kodaira A, Koizumi H, Hiraba H, Takeuchi Y, Koike M, Shimoe S. Bonding of resin luting materials to titanium and titanium alloy. J Oral Sci 2022; 64(3):181–4.

16. Lima VP, Soares K, Caldeira VS, Faria-E-Silva AL, Loomans B, Moraes RR. Airborne-particle Abrasion and Dentin Bonding: Systematic Review and Meta-analysis. Oper Dent 2021; 46(1):E21-E33.

17. Alseddiek A, Alzordok W, Attia A. Retention of hybrid-abutment-crowns with offset implant placement: influence of Crown materials and Ti-base height. BMC Oral Health 2023; 23(1):784.

18. Denkena B, Hurschler C, Bergmann B, Legutko B, Gustav M, Welke B. Influence of surface topography on junction strength of modular hip endoprostheses. Procedia CIRP 2022; 110:64–9. Available from: URL: https://www.sciencedirect.com/science/article/pii/S2212827122007958

19. Rehmer A, Bishop NE, Morlock MM. Influence of assembly procedure and material combination on the strength of the taper connection at the head-neck junction of modular hip endoprostheses. Clin Biomech (Bristol, Avon) 2012; 27(1):77–83.

20. Jauch-Matt SY, Miles AW, Gill HS. Effect of trunnion roughness and length on the modular taper junction strength under typical intraoperative assembly forces. Med Eng Phys 2017; 39:94–101.

21. Krull A, Morlock MM, Bishop NE. The Influence of Contamination and Cleaning on the Strength of Modular Head Taper Fixation in Total Hip Arthroplasty. The Journal of arthroplasty 2017; 32(10):3200–5.

22. Danoff JR, Longaray J, Rajaravivarma R, Gopalakrishnan A, Chen AF, Hozack WJ. Impaction Force Influences Taper-Trunnion Stability in Total Hip Arthroplasty. The Journal of arthroplasty 2018; 33(7S):S270-S274. Available from: URL: https://pubmed.ncbi.nlm.nih.gov/29428467/.

Figure 1

Click here to access/download;Figure;Figure 1.png

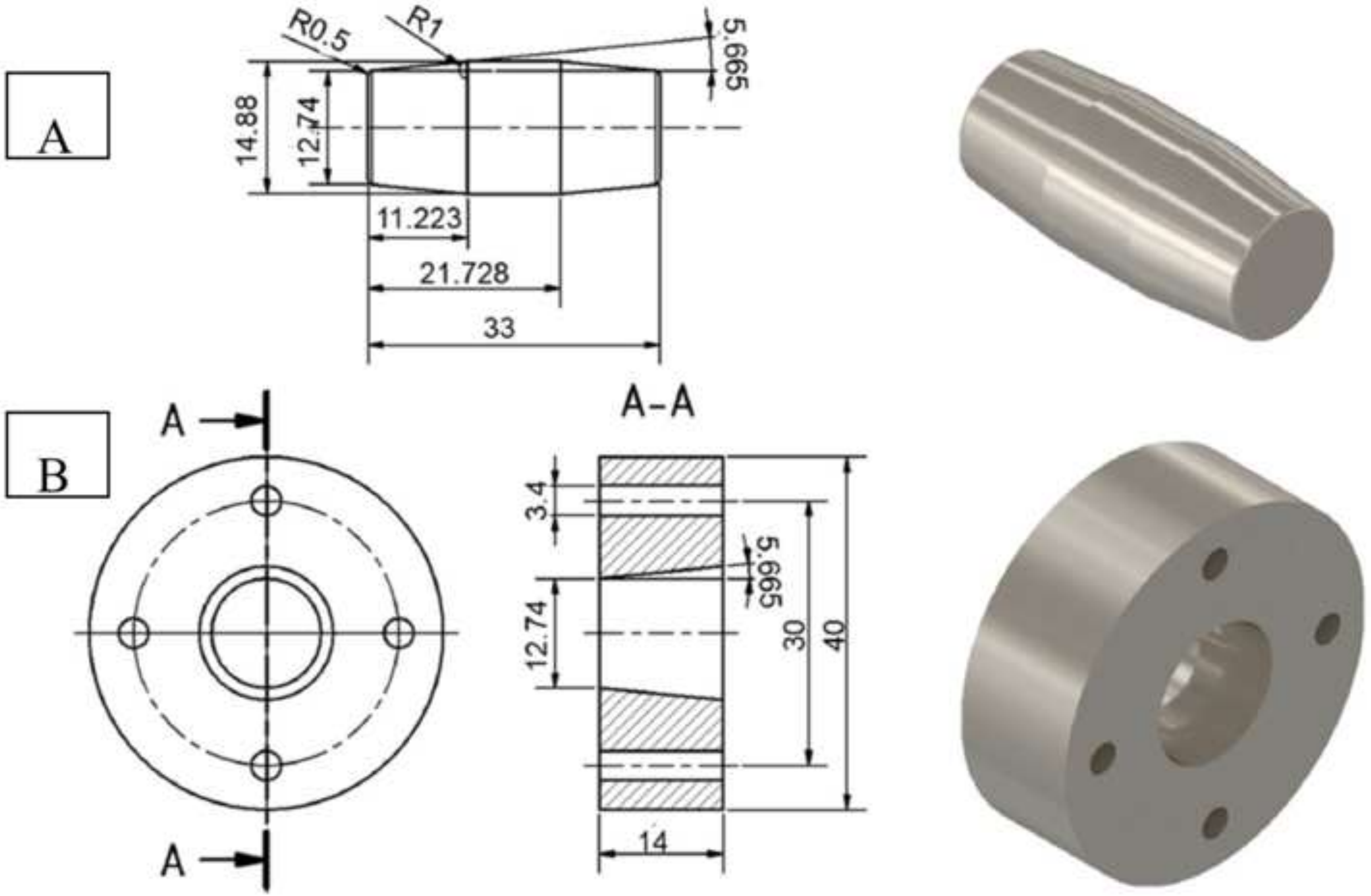

Figure 2

Click here to access/download;Figure;Figure 2.png

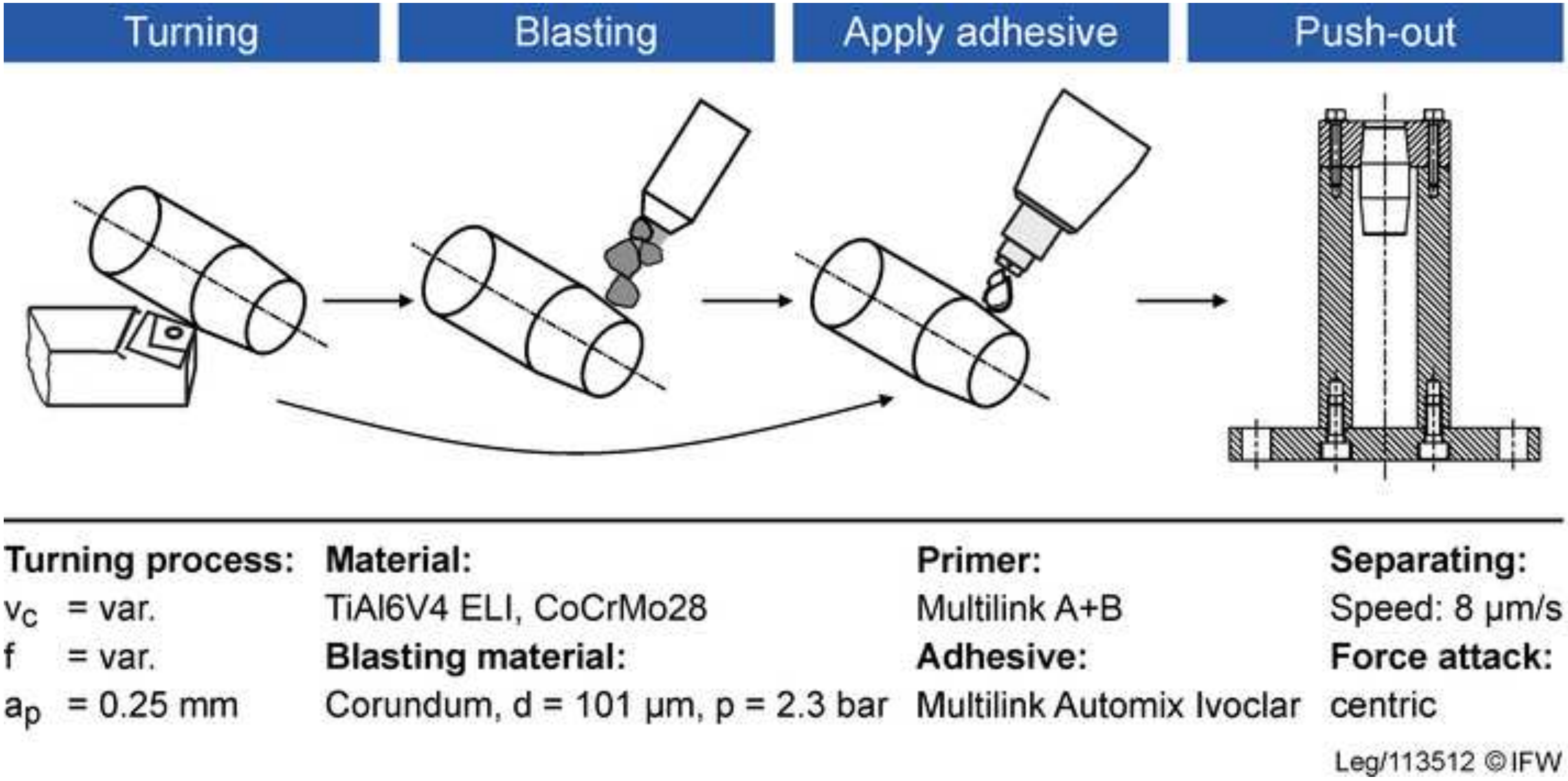

Figure 3

Click here to access/download;Figure;Figure 3.png

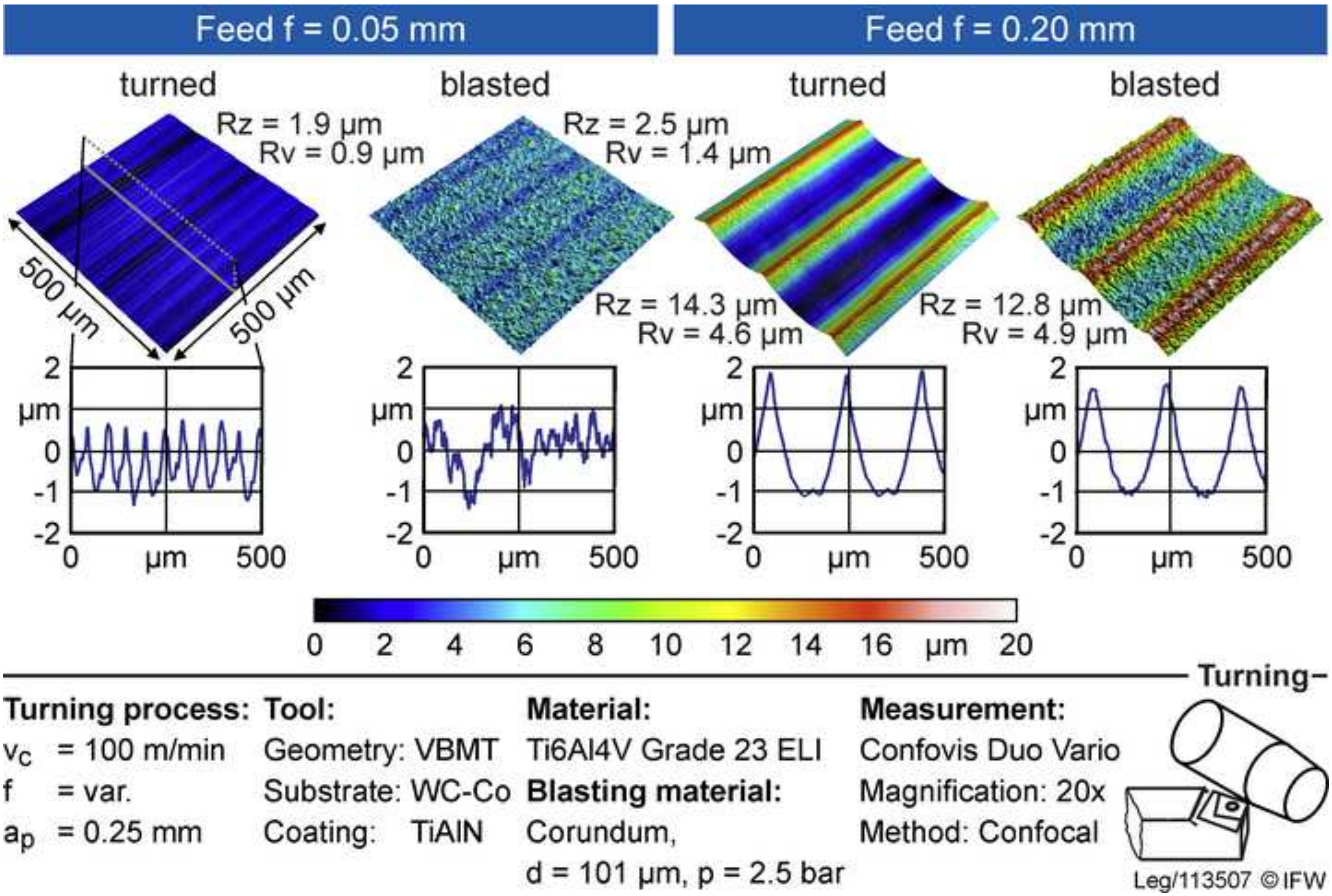

Figure 4

Click here to access/download;Figure;Figure 4.png

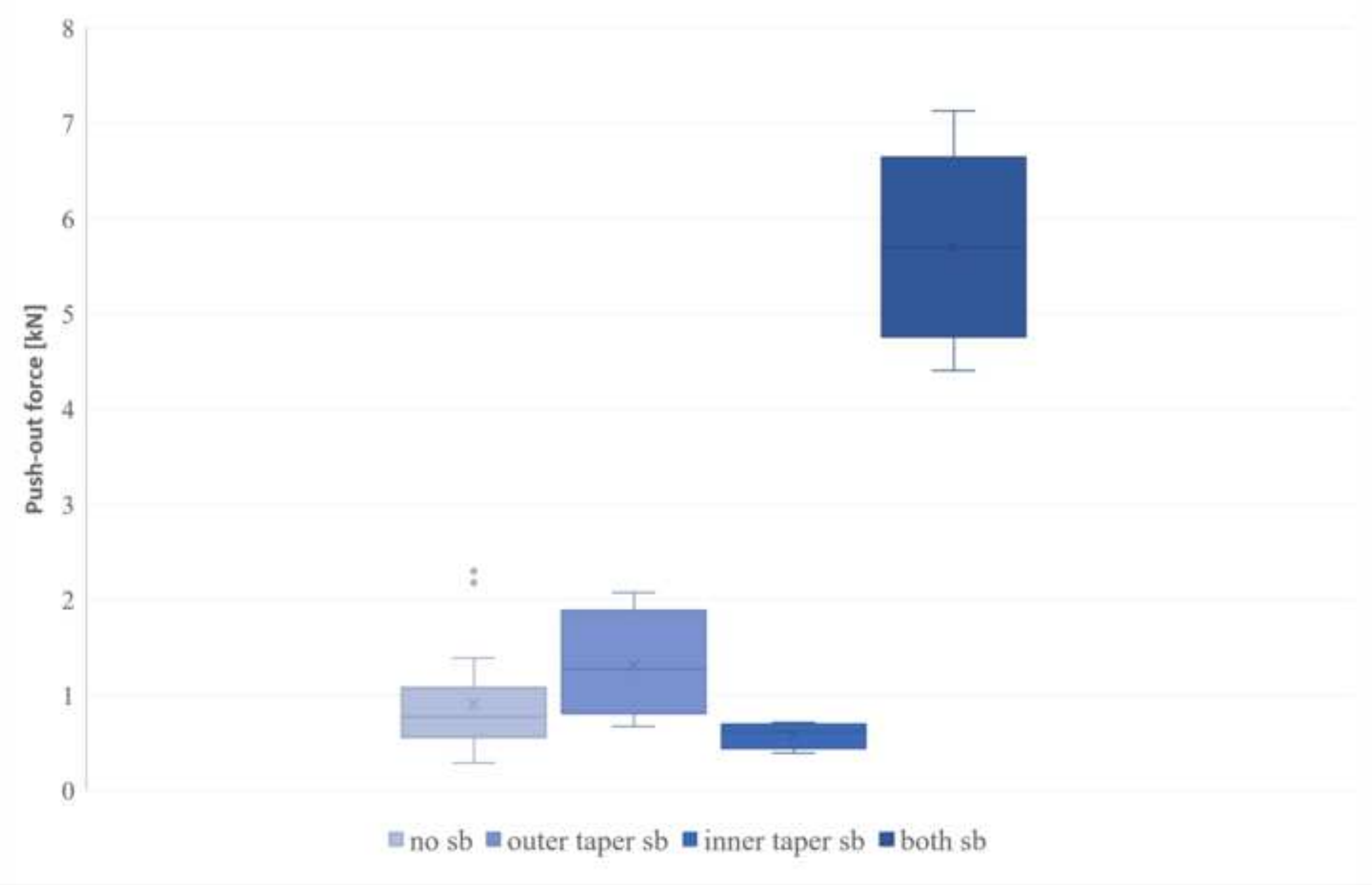

Figure 5

Click here to access/download;Figure;Figure 5.png

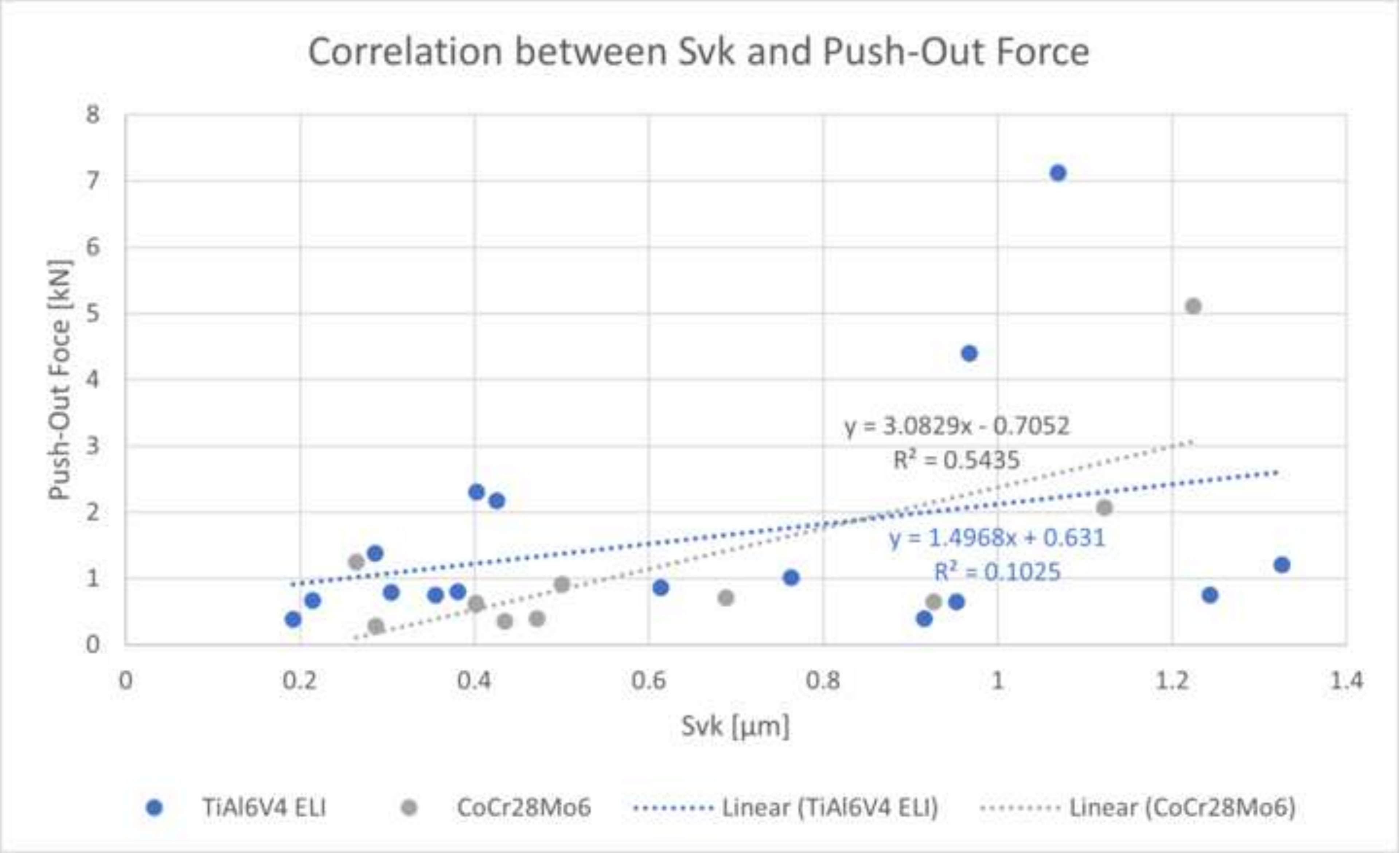

Figure 6

Click here to access/download;Figure;Figure 6.png

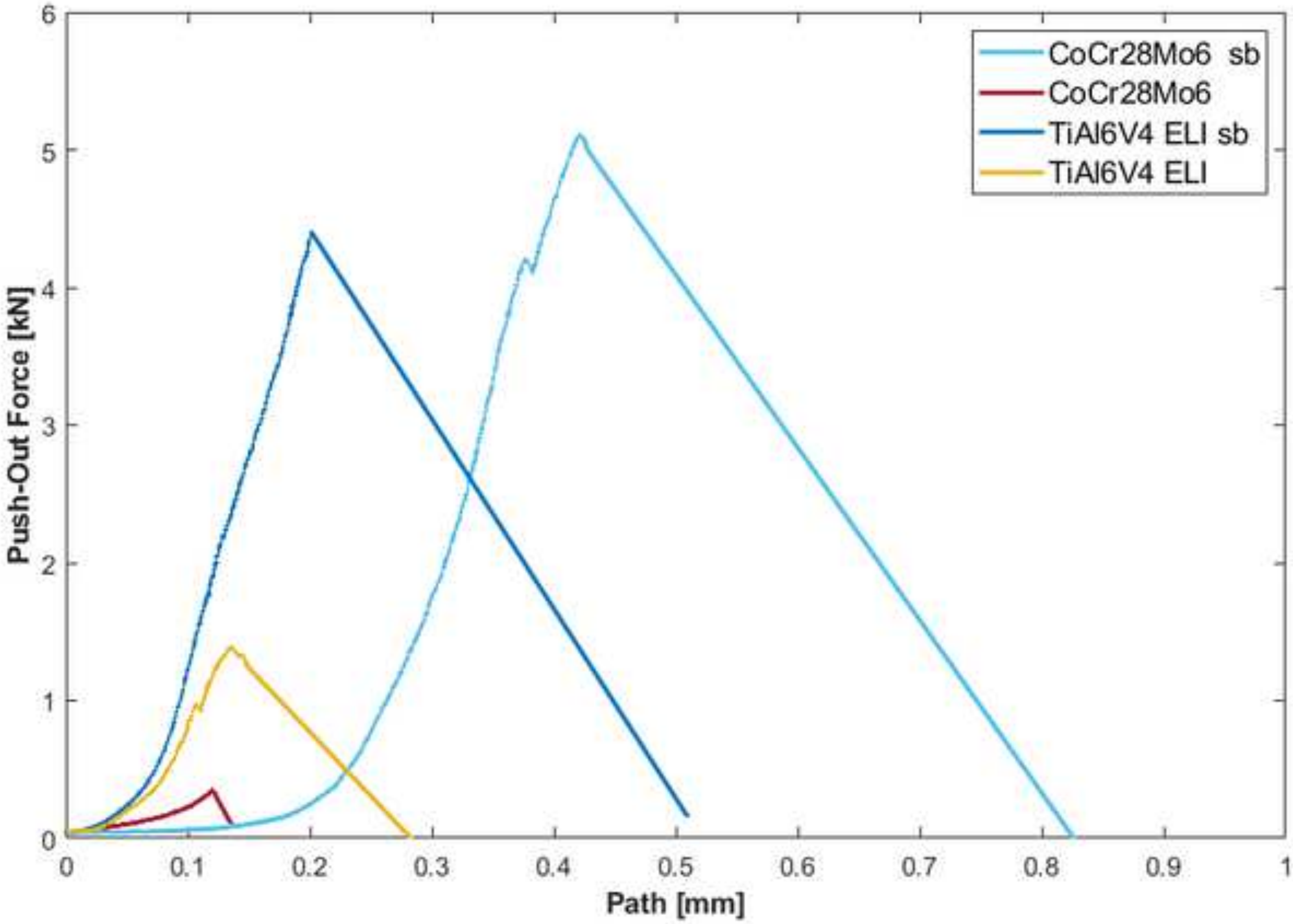

Click here to access/download
Supporting Information
force_displacement_data.xlsx

Supporting Information 2

Click here to access/download
Supporting Information
taper_geometry.xlsx

Supporting Information 3

Click here to access/download
Supporting Information
Table Taper Mismatch Supplemental.docx

Influence of the use of an adhesive connection on the joint strength of modular hip endoprostheses

Ann-Kathrin Einfeldt[a], Beate Legutko[b], Philipp-Cornelius Pott[c], Benjamin Bergmann[b], Berend Denkena[b], Christof Hurschler[a], Bastian Welke[a]*

[a] Laboratory for Biomechanics and Biomaterials, Department of Orthopedic Surgery – Annastift DIAKOVERE, Hannover Medical School, Anna-von-Borries-Str. 1-7, 30625 Hannover, Germany

[b] Institute of Production Engineering and Machine Tools, Leibniz University Hannover, An der Universität 2, 30823 Garbsen, Germany

[c] Department of Prosthetic Dentistry and Biomedical Materials Research, Hannover Medical School, Carl-Neuberg Str. 1, 30625 Hannover, Germany

*Corresponding author:

Bastian Welke (BW)

Welke.bastian@mh-hannover.de

ORCID ID: 0000-0003-2526-8129

Abstract

Introduction

Modular hip implants enables a more precise adaptation of the prosthesis to the patient's anatomy. However, they also carry the risk of increased revision rates due to micromotion at the taper junction. In order to minimize this risk, one potential solution is to establish an adhesive bond between the metal taper junctions. Load-stable bonding techniques, already successfully employed in dentistry for connecting materials such as metals and ceramics or different alloys, offer a promising approach. Nevertheless, the bond strength of tapered adhesive bonds in modular hip implants has not been investigated to date.

Materials and Methods

Twenty-eight tapered junctions, consisting of a taper (female taper) and a trunnion (male taper) were turned using TiAl6V4 ELI (n = 16) and CoCr28Mo6 (n = 12). The process parameters cutting speed (v_c = 50 m/min or 100 m/min) and feed (f = 0.1 mm, 0.05 mm or 0.2 mm) were varied for the trunnions. For each set of process parameters, one trunnion and one taper were additionally subjected to sandblasting. To investigate the effect of geometry, angular mismatch in the samples were measured. The taper pairs were bonded with a biocompatible adhesive, and push-out tests were subsequently performed.

Results

The push-out forces generated from the taper connections where both tapers were sandblasted showed a mean push-out force of 5.70 kN. For the samples with only the trunnion sandblasted, the mean force was 0.58 kN, while for the samples with only taper sandblasted the mean push-out force was 1.32 kN. When neither of the tapers was sandblasted the mean push-out force was 0.91 kN. No significant effect of the process parameters on the push-out force was observed. Only the reduced valley depth Svk showed a slight correlation for the CoCr28Mo6

samples (R^2 = 0.54). The taper pairs with taper mismatch (between trunnion and taper) greater than |0.1°| did not show lower push-out forces than the specimens with lower taper mismatch.

Conclusions

Sandblasted and adhesive-bonded tapered connections represent a viable suitable alternative for modular hip implant connections. Slight differences in taper geometry do not result in reduced push-out forces and are compensated by the adhesive. In mechanically joined tapers these differences can lead to higher wear rates. Further investigation under realistic test conditions is necessary to assess long-term suitability.

Keywords: modular hip endoprostheses, adhesive connection, junction strength, taper junction

Introduction

Total hip arthroplasty (THA) is one of the most common inpatient surgeries, with approximately 240,000 procedures performed each year [1]. Since the late 1990s, modular hip endoprostheses have allowed surgeons to better address individual patient characteristics by using specific implant components with different stem neck sizes and angles (1, 2)]. For example, bi-modular tapered neck adapters allowed the generation of different antetorsion and caput-collum-diaphyseal (CCD) angles, facilitating optimal reconstruction of the biomechanics of the joint (1)].

Nonetheless, the advantages of bi-modularity were associated with increased complications, resulting in revision rates of 6% compared to 3% for monolithic prosthesis systems after 5 years (3)]. Typical damage observed is the clinical failure of tapered stem junctions due to micromotion, which appear to be dependent on the design and material coupling (Co-Cr29-Mo vs. Ti-6Al-4V) at the junction between the stem and the adapter (2, 4)]. For instance, titanium neck adapters exhibited significantly larger micromotions than cobalt-chromium adapters and

contaminated interfaces also showed significantly larger micromotions (4)]. These micromotions can lead to fretting and crevice corrosion at the taper junction, potentially causing premature connection failure accounting for 2% to 3% of all THA revisions (5, 1)].

Despite being a well-known phenomenon the exact causes of theses complications remain poorly understood. However, a number of mechanisms and possible causes are commonly cited, such as galvanic and crevice corrosion, micromotions and fretting, and contamination of the joint leading to the release of metal ions (6)]. Furthermore, modularity plays a central role in the damage tolerance of implants, and each additional modular connection therefore increases the effect.

Another aspect leading to higher wear rates during dynamic loading is a so-called taper mismatch (7, 8)]. It describes the difference of the angle between the trunnion and the taper of the hip implant and is sometimes designed into the systems to ensure consistent performance (8)]. Nevertheless, a few finite element studies found out that smaller mismatches result into less wear in modular hip implants (7, 8)]. Taper mismatch can be negative (larger taper than trunnion), or positive (smaller taper than trunnion). Ahkanfar et al. performed a comparative FEM study of the effect of different taper mismatch angels and suggested that taper mismatch should be manufactured with to less than 6’ or 0,1° (9)].

The above mentioned possible causes of taper junction failure area multiple and have diverse causes. Despite their utility in restoring patient specific geometries, bi-modular hip implants have widely been recalled from the market due to the relatively high rates of failure observed. One possible method to address the aforementioned failure mechanisms (micromotion, fretting-corrosion, contamination, and taper-missmatch tolerances) may be to establish an adhesive bond in the metal taper junctions. Such an adhesive bond has the potential to enhance the mechanical load transfer between the metal taper partners and to prevent corrosion and release of particles and ions into the joint. These types of adhesively joined interfaces are not new. In

dentistry, adhesive techniques have been well established for bonding various materials in different types of dentures and crowns (10–13)]. Thus, titanium and cobalt-chromium tapered bonds have successfully been utilized and investigated in this context (14, 15)]. Furthermore, several bonding systems have been reported and the use of adhesives proven to be effective for bonding titanium alloys (15)]. Bonding systems investigated in this context have included different primers and luting agents, often in conjunction with surface modification such as airborne-particle abrasion, a specialized surface treatment technique similar to sandblasting (16)]. The dental application most comparable to the taper-junctions addressed in this study are hybrid abutments, where the respective restoration is bonded to standardized implant abutments (17)]. However, the bond strength of tapered adhesive bonds as used in in modular hip implants has not been investigated to date. Due to the high revision rates in bi-modular hip implants, they widely disappeared from the market. Nevertheless, this study aims to investigate adhesive bonding with the aim of addressing the issues that led to their failure. The aim of this study was therefore to perform an exploratory investigate of the possible suitability of using an adhesive bond to join modular hip implant junctions. To achieve this goal, various process parameters and the resulting surface topographies of titanium and cobalt chromium bonded joints were examined in relation to their bond strength.

Materials and Methods

Specimen preparation

In total 28 taper connections consisting of a trunnion (male taper) and a taper (female taper) were manufactured. The tapers were manufactured on a linear lathe (CTX 420, Gildemeister, Wernau, Germany) and the trunnions on the turnmill center (NTX 1000, DMG MORI, Wernau, Germany). For turning the trunnion, indexable inserts (type VBMT 1604 MM 1115, Sandvik Tooling Deutschland GmbH, Duesseldorf, Germany) were used. The trunnion has a nominal taper angle of θ_{AK} = 11.33°, as well as the diameters of d_E = 12.74 mm and d_H = 14.88 mm. The

length of the angular surface in relation to the central axis of the trunnion was l_{AK} = 11.22 mm and the length of the bore of the taper was l_{IK} = 14 mm to ensure that in case of minor deviations of the geometry due to the manufacturing process, the entire taper length is still in contact at the junction.

The process parameters cutting speed (v_c = 50 m/min or 100 m/min) and feed (f = 0.1 mm, 0.05 mm or 0.2 mm) were varied for the trunnions, resulting in a total of seven different testing groups, each consisting out of four taper pairs (table 1). In the first four groups the taper pairs (Ti 1 – Ti 4) were made out of TiAl6V4 ELI whereas in the remaining three groups the samples were manufactured out of CoCr28Mo6 (CoCr 5-CoCr 7). The depth of cut a_p = 0.25 mm were kept constant. The process parameters for the tapers were not varied to ensure that all observed effects were dependent on the surface topography of the trunnions. The tapers were manufactured with indexable inserts (type CCMT 060204 Sandvik Tooling Deutschland GmbH, Duesseldorf, Germany) on a boring bar. The machining process was performed with v_c = 70 m/min, f = 0.1 mm and ap = 0.25 mm. In each testing group one trunnion and one taper were additionally sandblasted (sb). In groups Ti 2, Ti 4, CoCr 5 and CoCr 7 both tapers of a taper pair were sandblasted whereas in the remaining three groups different tapers were sandblasted (table in supplemental material). The sandblasting pressure was set to p = 2.3 bar, and the taper contact surface was manually blasted with a grain size of x = 101 µm corundum at approximately 10 mm distance from the surface for 30 seconds.

Table 1: Overview over the different process parameters for each taper group. Tapers in group 1-4 were manufactured from TiAl6V4 ELI, tapers in group 5-7 were manufactured from CoCr28Mo6 (shaded in grey).

	**v_c [m/min]**	**f [mm]**
Ti 1	50	0.10

Ti 2	100	0.05
Ti 3	50	0.05
Ti 4	100	0.20
CoCr 5	50	0.10
CoCr 6	100	0.05
CoCr 7	50	0.05

Measurement of the samples

As possible angular mismatch of the tapers can also influence the separating forces that occur in the taper connections, a 3D coordinate measurement system (Reference Xi 1076, Leitz, Stockholm, Sweden) was used to determine the resulting geometry of the manufactured tapers.

Subsequently, roughness measurements were performed for the trunnion and tapers. The tapers were scanned over a distance of 8 mm (MarSurf LD 130 with the LP C 3.5-102/90° probe, Mahr, Göttingen, Germany). The probe then repeated this process a further nine times at different starting points in order to obtain a more accurate representation of the surface topography across a wider measuring distance.

The roughness measurement of the trunnions was conducted using an optical measuring device over a length of 5 mm (Duo Vario, Confovis, Neu-Isenburg, Germany). Measurements were taken for all tapers, starting from the main diameter in the direction of the end diameter and from the end diameter in the direction of the main diameter. This approach resulted in a total measuring length of 10 mm per taper, allowing for the analysis of potential deviations in the surface over the entire contact area of the taper.

The measurement data was analysed using the MountainsMap analysis programme (Digital Surf, 8.2, Besançon, France). The characteristic values of the surface topographies were determined. The roughness parameters maximum height of profile (Rz) and maximum depth of

profile (Rv) according to DIN EN ISO 21920 and the primary surface topography profile (Reduced Peak Hight (Spk), Reduced Valley Depth (Svk)) according to DIN EN ISO 25178, were recorded over a length ranging from 3.8 to 4.6 mm.

Bonding the tapers

After manufacturing and blasting, the tapers were bond with a biocompatible adhesive system. The adhesive is a self-curing luting composite used for the adhesive luting of metal and metal-ceramics, among other materials. The adhesive system used (Multilink Automix, Ivoclar Vivadent AG, Ellwangen, Germany) is a self-curing material consisting of a 2-component primer system (Multilink Primer A + B) and a 2-component luting resin. Before bonding, the tapers were cleaned in an ultrasonic bath with alcohol to eliminate any negative influences of possible contamination on the bond.

The two primer components A and B were mixed in a 1:1 ratio and evenly applied to the contact surfaces without the formation of drops until the surface was completely wetted. The exposure time for the primer was 2 minutes. The surfaces that needed to be free of the luting resin were isolated with cling film. The luting resin was then applied bubble-free into the opening of the taper using the application cannula and the tapers were joined together (Figure 2). The trunnion was inserted into the taper and allowed to sag under its own weight until the adhesive oozes out at the edge of the contact surface. This excess adhesive can be removed within the curing time of 8 to 10 minutes at 23°C ± 1°C (Figure 2).

Figure 2: Schematic illustration of the individual processing steps and the setup for the push-out tests

Push-out tests

The push-out tests were performed using a universal testing machine (8501, Instron, Illinois Tool Works Inc., Norwood, USA). The tests were carried out after a curing time of 24 hours at 23°C ± 1°C of the probes. The machine's stamp moved down hydraulically at a velocity of 0.48 mm/min, pressing the trunnion out of the taper. At every 0.001 mm displacement of the hydraulic cylinder, the following parameters were recorded: time, axial force and axial displacement. The measured displacement of the testing machine was subsequently corrected for the rigidity of the setup.

Results

Taper geometry

The main diameter of the trunnion was targeted to be $d_M = 14.88$ mm, the end diameter $d_E = 12.74$ mm, the taper length l = 11.22 mm, and the angle θ_n = 11.33°.

All trunnions show deviations from the nominal manufacturing values, with a mean deviation of all trunnions was 0.18 ± 0.19 mm, whereas the Ti 3-row showed the smallest deviations (Deviation d_M = 0.06 mm). The TiAl6V4 ELI tapers tend to be above, and the CoCr28Mo6 tapers below the nominal values. The mean deviation of the end diameter for all trunnions was 0.22 ± 0.23 mm, and for the taper length 0.03 ± 0.03 mm. The Ti 2-row shows the smallest deviations from the nominal value for both parameters (Deviation d_E = 0.04 mm, Deviation l = 0.004 mm). CoCr28Mo6 tapers showed greater dispersions than the TiAl6V4 ELI tapers. The mean angle deviation of all trunnions is 0.04 ± 0.02°, with the Ti 3-row again showing the smallest deviations (θ = 0.02°) from the nominal manufacturing value whereby and the CoCr28Mo6 tapers exhibiting greater deviations than the TiAl6V4 ELI tapers.

The face diameter of the taper was aimed to be d_F = 14.88 mm, the end diameter d_E = 12.74 mm and the angle θ_n = 11.33°.

All face diameters of the tapers are above the nominal value (Deviation d_F = 0.11 ± 0.02 mm) whereas in the end diameter they are below the nominal value (Deviation d_E = 0.37 ± 0.02 mm). The mean deviation of the nominal angle of all tapers is 0.64 ± 0.02° and is above the nominal value for all tapers.

Due to the deviations from the nominal values different free volumes - areas where there is no contact between the tapers - exist for the taper pairs. All 28 taper pairs exhibit a negative taper mismatch (trunnion angle < taper angle) and therefore a proximal contact. All taper pairs show a taper mismatch greater than |0.1°| with a mean of -0.182 ± 0.047° (range -0.012° to -0.369°) (table in supplemental material).

Roughness

The roughness measurement of the trunnions after sandblasting shows the greatest roughness in the Ti 4-group, followed by the CoCr 5 and Ti 1 groups (Table 2). CoCr28Mo6 tapers exhibit greater Rz and Rv values than TiAl6V4 ELI tapers when the same process parameters are used. Ti 4 group reaches the greatest Spk, followed by the Ti 1 and the CoCr 5 group. CoCr 5 group show the greatest Svk.

Table 2: Overview over the different roughness and process parameters for each sample group of trunnions before (shaded in blue) and the one trunnion per sample group after sandblasting; Legend: Rz = maximum height of profile; Rv = maximum depth of profile; Spk = Reduced Peak Hight; Svk = Reduced Valley Depth

	**Mean Rz [µm]**	**Mean Rv [µm]**	**Mean Spk [µm]**	**Mean Svk [µm]**	**Rz [µm]**	**Rv [µm]**	**Spk [µm]**	**Svk [µm]**
Ti 1	4.812	2.335	1.218	0.685	4.531	2.014	1.437	0.932
Ti 2	1.892	0.968	0.330	0.396	2.027	1.118	1.054	0.983
Ti 3	2.412	1.221	0.615	0.394	1.495	0.817	1.004	1.085

Ti 4	15.080	5.470	8.554	2.326	12.500	4.705	6.625	1.115
CoCr 5	5.057	2.673	0.837	1.316	6.595	4.164	0.798	3.035
CoCr 6	2.606	1.274	0.475	0.375	2.175	1.090	1.010	0.744
CoCr 7	2.854	1.508	0.480	0.689	2.488	1.515	0.813	1.331

After sandblasting, the trunnions show a more porous surface. For the TiAl6V4 ELI tapers the Rz and Rv values hardly change whereas for the CoCr28Mo6 tapers these values were reduced after sandblasting. Both the Spk and the Svk increases after sandblasting for both materials.

As there was no variation in the process parameters for the tapers, only differences between the sandblasted and not sandblasted tapers were evaluated. The sandblasted surface is more porous (Figure 3, Supplement) leading to increased roughness parameters compared to the untreated surface.

Figure 3: Surface and profile plots of a turned sample (left in each case) and a turned and subsequently sandblasted sample (right in each case) of a trunnion.

Push-out tests

The results of the push-out tests show the greatest forces ($mean_F$ = 5.70 kN) for the taper connections where both tapers were sandblasted (Figure 4, Supplement). All other pairing situations show smaller forces (only taper sandblasted $mean_F$ = 1.32 kN, no sandblasting $mean_F$ = 0.91 kN), with the smallest force when only the trunnion was sandblasted (meanF = 0.58 kN).

Figure 4: Results of the push-out test for all taper connections and materials divided into groups by the sandblasting (sb) situation. Values above and below 1.5 times the interquartile range are shown as outliers.

To investigate whether the mismatches in taper geometry influence the resulting push-out forces the free volume for each taper pair was calculated. The calculated free volume includes the taper mismatch and the roughness profile resulting from the machining process of the trunnion and the taper. No correlations between free volume and therefore the adhesive volume and the push-out forces could be seen.

When looking at the push-out forces in relation to the different process parameters and therefore the surface topographies, no correlation between the roughness parameters Rz and Rv and the push-out force were observed ($R^2_{Rz} = 0.03$; $R^2_{Rv} = 0.08$).

No correlations were observed ($R^2_{allsamples} = 0.02$, $R^2_{Ti} = 0.02$, $R^2_{CoCr} = 0.13$) for the push-out forces in relation to the tip height. For the Svk values, a slight correlation ($R^2_{CoCr} = 0.54$, $p = 0.08$) can be observed especially for the CoCr28Mo6 samples (Figure 5, Supplement).

Figure 5: Correlation between Svk (Reduced Valley Depth) and push-out force for all TiAl6V4 ELI samples (blue) and CoCr28Mo6 samples (grey). Dotted line shows the resulting regression line for each material type.

The force-distance curves of the push-out tests are exemplarily shown for individual samples of each group (see Fig 6). The graph shows the smallest incline for the only turned CoCr28Mo6 sample (Fig. 6, red line). For the only turned TiAl6V4 ELI sample the incline is steeper (Fig. 6, yellow line). When both tapers of one sample are sandblasted the steepest inclination profiles

are seen, (Fig. 6, light and dark blue). When one sample is sandblasted and one sample is turned, no clear inclination profile can be observed for the samples.

Figure 6: Force-Path curves of the push-out tests exemplarily shown for individual samples of the different groups (CoCr28Mo6 only turned = red; TiAl6V4 ELI only turned = yellow; CoCr28Mo6 both sandblasted = light blue; TiAl6V4 ELI both sandblasted = dark blue. The measured displacement of the testing machine was corrected for the stiffness of the setup.

A visual analysis of the tapers after the push-out tests showed, that the adhesive adheres only to the sandblasted surfaces. No adhesive residues were observed macroscopically at the surfaces that were only turned.

Discussion

Taper geometry

The smallest deviations in taper geometry could be observed when the cutting speed and the feed were both small as shown in the Ti_3 group with a d_M = 0.06 mm (v_c = 50 m/min and f = 0.05 mm). The feed seems to have a greater influence on the precision of the taper geometry than the speed, as both groups with a feed of 0.05 mm (Ti_2 and Ti_3) show the smallest deviations from nominal values even though different speeds were used. CoCr28Mo6 trunnions tend to be below the nominal values whereas TiAl6V4 tend to be above them. The tapers show that the face diameter of all tapers lies above whereas the end diameter lies below the nominal value. This is due to the different tool deflection caused by the different material properties.

When comparing the taper material, CoCr28Mo6 shows greater deviations from the nominal values than TiAl6V4 ELI. As CoCr28Mo6 is harder than TiAl6V4 ELI, this could be the reason. Overall, the results show that the material has a greater influence on the deviations on taper geometry than the process parameters.

All 28 samples show a proximal contact area. Bitter et al. (8) recommend that a distal contact area should be chosen to reduce the wear rate. As there only samples showing a proximal contact area no conclusion can be drawn about which contact area is to be preferred in adhesive bonded taper pairs.

All taper pairs show a taper mismatch greater than |0.1°|. The results of the push-out tests indicate that there is no correlation between the amount of mismatch and the push-out force. Therefore, the adhesive appears to compensate slight differences in taper geometry and consequently taper mismatch.

Roughness

Most stem tapers that are turned have trough to peak heights form 10 to 13 µm. In order to investigate the influence of the surface topographie on the push-out force the range of the surface roughness is from Rz = 5 µm to Rz = 15 µm. CoCr28Mo6 was observed to have greater Rz and Rv roughness than TiAl6V6 ELI. Reduced peak height on the other hand was greater for Ti than for CoCr28Mo6. Before sandblasting, it could be shown that the feed has the greatest influence on the roughness, whereas the speed does not seem to influence this parameter.

After sandblasting, the roughness parameters hardly change for the TiAl6V6 ELI trunnions whereas for the CoCr28Mo6 tapers roughness is reduced. It cannot be ruled out that this difference is due to the manual sandblasting, which might have led to inconsistent influences on the surface. When looking at the surface topographies before and after sandblasting it is clear that the surface topography after sandblasting is more stochastic and the possible bonding surface is enlarged. For tapers the roughness increases after sandblasting regardless of the material.

Push-out tests

A preliminary study on taper junctions of the same geometry and materials showed that tapers with an assembled at a speed of v = 8 µm/s and a force of up to F = 4kN reached push-out forces between $F_{separate}$ = 0.79 and 1.8 kN (18). The push-out tests in this study show that taper connections where both tapers were sandblasted before bonding have the greatest push-out forces, ranging from 4.4 to 7.1 kN. These adhesively bonded junctions thus exceeded native taper junctions by a minimum of 244 %. Other studies of native taper pull out force have reported ranges of pull-off force under normal conditions for different material pairings and surface modifications ranging from 1.6kN to 3kN (19–21), a study using very high impaction forces of 14kN reported pull-off forces of up to 6kN (22). Mechanically, the push-out test we performed is equivalent to the pull off test as described in the cited studies.

The Svk shows a slight correlation with the resulting push-out force especially for the CoCr28Mo6 tapers whereas all other roughness parameters are not showing any correlations. The greater the Svk the more liquid and in this case, adhesive, can be collected on the surface of a sample. A greater amount of adhesive on the surface therefore leads to increased bonding strength. After sandblasting this parameter increases for all the samples. This is in accordance with the increased push-out forces for the sandblasted taper connections. Also, the force-displacement curves and the visual analysis of the tapers support this thesis. The steeper inclination of the force time curve for the sandblasted samples, indicate a stiffer junction of the taper pairs as the adhesive adheres to both taper partners. Sandblasting leads to a more porous surface where the adhesive is more likely to adhere to than to only turned samples. This could be seen in the visual analysis after the push-out test.

Even though the other roughness parameters did not influence the push-out forces, connections where only the taper was sandblasted show higher push-out forces than taper connections without sandblasting or with only trunnion blasting. As the sandblasted tapers exhibit increased roughness parameters, a slight influence of the roughness of sandblasted tapers can be assumed.

Nevertheless, the greatest bonding strength is achieved when both tapers are sandblasted, which is why further investigations should be conducted in this manner. In further investigations, adhesive bonded taper connections should be tested under more realistic conditions. Micromotion is the most common cause of failure of modular implant connections. These possible micromotions were not investigated in our study. This is the subject of further planned fatigue tests with 10 million load cycles under standardised testing conditions.

In the current study, we investigated taper combinations consisting of both partners being either TiAl6V6 ELI or CoCr28Mo6. Future investigations should also test the combination of these two materials. Furthermore, further research will investigate possible methods of revising of glued taper junctions.

Conclusion

The push-out forces generated from the sandblasted tapers resulted in the highest push-out forces and were greater than push-out forces from mechanical assembled taper pairs reported in the literature. Therefore, the conclusion can be drawn that sandblasted and adhesive bonded taper connections are promising alternative for modular hip implant junctions. Slight differences in taper geometry are compensated by the adhesive and do not influence the push-out forces. Further investigation under realistic testing conditions is necessary to assess the suitability in the long-term.

List of abbreviations

CCD:	caput-collum-diaphyseal
f:	feed
Rv:	maximum depth of profice

Rz:	maximum height of profile
Sb:	sandblasted
Spk:	reduced peak hight
Svk:	reduced valley depth
THA:	Total hip arthroplasty
v_c:	cutting speed

References

1. Grupp TM, Weik T, Bloemer W, Knaebel H-P. Modular titanium alloy neck adapter failures in hip replacement--failure mode analysis and influence of implant material. BMC Musculoskelet Disord 2010; 11:3.

2. Jauch SY, Huber G, Haschke H, Sellenschloh K, Morlock MM. Design parameters and the material coupling are decisive for the micromotion magnitude at the stem-neck interface of bi-modular hip implants. Med Eng Phys 2014; 36(3):300–7.

3. National Joint Replacement Registry, Australian Orthopaedic Association. 2018 Hip, Knee & Shoulder Arthroplasty Annual Report; 2018.

4. Jauch SY, Huber G, Hoenig E, Baxmann M, Grupp TM, Morlock MM. Influence of material coupling and assembly condition on the magnitude of micromotion at the stem-neck interface of a modular hip endoprosthesis. J Biomech 2011; 44(9):1747–51.

5. Jauch SY, Huber G, Sellenschloh K, Haschke H, Baxmann M, Grupp TM et al. Micromotions at the taper interface between stem and neck adapter of a bimodular hip prosthesis during activities of daily living. J Orthop Res 2013; 31(8):1165–71.

6. Gilbert JL, Buckley CA, Jacobs JJ. In vivo corrosion of modular hip prosthesis components in mixed and similar metal combinations. The effect of crevice, stress, motion, and alloy coupling. J Biomed Mater Res 1993; 27(12):1533–44.

7. Gustafson JA, Mell SP, Levine BR, Pourzal R, Lundberg HJ. Interaction of surface topography and taper mismatch on head-stem modular junction contact mechanics during assembly in modern total hip replacement. J Orthop Res 2023; 41(2):418–25. Available from: URL: https://pubmed.ncbi.nlm.nih.gov/35488727/.

8. Bitter T, Khan I, Marriott T, Lovelady E, Verdonschot N, Janssen D. The effects of manufacturing tolerances and assembly force on the volumetric wear at the taper junction in modular total hip arthroplasty. Comput Methods Biomech Biomed Engin 2019; 22(13):1061–72.

9. Ashkanfar A, Langton DJ, Joyce TJ. A large taper mismatch is one of the key factors behind high wear rates and failure at the taper junction of total hip replacements: A finite element wear analysis. Journal of the mechanical behavior of biomedical materials 2017; 69:257–66. Available from: URL: https://pubmed.ncbi.nlm.nih.gov/28110182/.

10. Khasawneh S, al-Wahadni A, Lloyd CH. Comparison of bond strengths between adhesive and conventional acrylic resins to cobalt chromium denture base alloy. Eur J Prosthodont Restor Dent 2003; 11(3):119–24.

11. Pott PC, Stiesch M, Eisenburger M. Influence of artificial aging on the shear bond strength of zirconia-composite interfaces after pretreatment with new 10-MDP adhesive systems. Journal of Dental Materials and Techniques 2016; 5(1).

12. Pott P-C, Syväri T-S, Stiesch M, Eisenburger M. Influence of nonthermal argon plasma on the shear bond strength between zirconia and different adhesives and luting composites after artificial aging. J Adv Prosthodont 2018; 10(4):308–14.

13. Kim D-S, Ahn J-J, Kim G-C, Jeong C-M, Huh J-B, Lee S-H. Influence of Non-Thermal Atmospheric Pressure Plasma Treatment on Retentive Strength between Zirconia Crown and Titanium Implant Abutment. Materials (Basel) 2021; 14(9).

14. Taira Y, Yanagida H, Matsumura H, Yoshida K, Atsuta M, Suzuki S. Adhesive bonding of titanium with a thione-phosphate dual functional primer and self-curing luting agents. Eur J Oral Sci 2000; 108(5):456–60.

15. Kodaira A, Koizumi H, Hiraba H, Takeuchi Y, Koike M, Shimoe S. Bonding of resin luting materials to titanium and titanium alloy. J Oral Sci 2022; 64(3):181–4.

16. Lima VP, Soares K, Caldeira VS, Faria-E-Silva AL, Loomans B, Moraes RR. Airborne-particle Abrasion and Dentin Bonding: Systematic Review and Meta-analysis. Oper Dent 2021; 46(1):E21-E33.

17. Alseddiek A, Alzordok W, Attia A. Retention of hybrid-abutment-crowns with offset implant placement: influence of Crown materials and Ti-base height. BMC Oral Health 2023; 23(1):784.

18. Denkena B, Hurschler C, Bergmann B, Legutko B, Gustav M, Welke B. Influence of surface topography on junction strength of modular hip endoprostheses. Procedia CIRP 2022; 110:64–9. Available from: URL: https://www.sciencedirect.com/science/article/pii/S2212827122007958

19. Rehmer A, Bishop NE, Morlock MM. Influence of assembly procedure and material combination on the strength of the taper connection at the head-neck junction of modular hip endoprostheses. Clin Biomech (Bristol, Avon) 2012; 27(1):77–83.

20. Jauch-Matt SY, Miles AW, Gill HS. Effect of trunnion roughness and length on the modular taper junction strength under typical intraoperative assembly forces. Med Eng Phys 2017; 39:94–101.

21. Krull A, Morlock MM, Bishop NE. The Influence of Contamination and Cleaning on the Strength of Modular Head Taper Fixation in Total Hip Arthroplasty. The Journal of arthroplasty 2017; 32(10):3200–5.

22. Danoff JR, Longaray J, Rajaravivarma R, Gopalakrishnan A, Chen AF, Hozack WJ. Impaction Force Influences Taper-Trunnion Stability in Total Hip Arthroplasty. The Journal of arthroplasty 2018; 33(7S):S270-S274. Available from: URL: https://pubmed.ncbi.nlm.nih.gov/29428467/.

Dear Dr. Antonio Riveiro Rodríguez,

Thanks a lot for the opportunity to revise our paper in order to resubmit it to PLOS ONE. We have worked on all comments and have implemented all of the suggestions and points of criticism. The present response is done in a stepwise way regarding the reviewer's comments (italic). Corresponding changes and additions have been highlighted in yellow in the "manuscript with track changes".

Reviewer #1: *The paper presents an interesting approach to secure taper connections in total hip arthroplasty against fretting and fretting-corrosion, respectively, via adhesive bonding of the connection. To do so, taper connections were fabricated, adhesively bonded and, subsequently, the resulting bonding strength was assessed in push-out tests. For inner tapers, a variety of different fabrication parameters was used which resulted in different surface topographies. The different topographies, however, did not influence the resulting bonding strength. In addition, one part or both parts of the taper connections were sandblasted, which influenced the resulting push-out forces.*

It was, however, not clearly addressed, which taper connection would benefit from adhesive bonding, since modularity (aside from the better adjustment options) also allows the replacement of individual parts of the endoprosthesis in case of a revision. This would in my opinion be compromised by adhesive bonding unless the bonding could be unglued in case of a revision (?). This should definitely be addressed in the discussion of the study.

Thank you very much for this important note. Our paper in fact focusses on the connection between the shank and a modular taper. Your comment regarding the replacement of the individual parts is really valuable and we added a section in the discussion (L347-350 in the revised manuscript).

Furthermore, it remained unclear to me, why so many different combinations processing parameters were used (Table 1 is rather confusing), especially since they did not really influence the resulting bonding strengths. Here, the paper would benefit from reducing the presented magnitude of different taper variations. Maybe the focus could be put on sandblasting vs. turned surfaces and the used materials?

Thank you very much for this valuable comment. We agree that the table was quite confusing. We have simplified the table and described the sample groups in the text more clearly (L130-140). We nonetheless think that investigating different process parameters provides valuable information and that interestingly the different process parameters did not have an influence on the bonding strength which we did not anticipate.

Major revisions are, therefore, necessary before publication of the study.

<u>*Some more specific comments are the following:*</u>

Introduction:

As already pointed out, it remains unclear, which taper connection should or could be addressed with adhesive bonding approach. In the introduction you refer to studies regarding dual-neck total hip endoprosthesis, but these have widely disappeared from the market, as far

as I know. The taper junction you actually tested appears to rather mimic the taper connection between head and stem taper (even though you used a twice as large taper angle).

Thank you very much for this note. As stated above, the paper focusses on the connection between the shank and a modular taper. We are aware of the disappearance of dual-taper junctions from the market because of the high revision rates. The motivation of this study was to investigate a possible improvement of the taper junctions which may allow dual-taper junctions to be safely used again, and in particular to exploit the flexibility in aligning the joint that they can provide in achieving an anatomically aligned joint reconstruction. We have supplemented some parts of the introduction to make this clearer to the reader.

Material and Methods:

Line 108: The taper angle is given as 5,665° but from Figure 1 it appears that this value refers to the half of the taper angle, which would result in a taper angle > 11°. Please be more accurate here and throughout the manuscript.

Thank you very much for this valuable comment. We have adjusted this accordingly so that the taper angle now has the correct value in the whole manuscript (L115-116).

The reviewer is absolutely right that the taper angle is >11°. We chose this larger angle to compare the results from this study with our preliminary study (18). In the preliminary study, we investigated the influence of the surface topography on the push-out force. The larger angle represents an unfavorable situation for the connection, both for the mechanical and for the bonded connection. We intentionally chose this in our project. Due to the larger taper angle, the average push-out force was only 1.8kN. In the current study, the same bonded taper junction achieved forces three times as high. In our view, this shows the potential of the bond.

Nevertheless, we are currently conducting a study in which we are investigating the cone connection with the taper angle of 12/14.

Line 114 -125: It is very hard for the reader to understand the combination of the process parameters used for manufacturing the specimens. In line 115 for example you write that two cutting speeds and three feeds were used, which would make six. From the table it becomes clear that not all parameters were combined with each other, but this does not become clear from the text. Also, it does not become clear, which material was used for which parameter combination and whether you produced inner and outer tapers from the two materials. Furthermore, it remains unclear how the different materials were combined for forming the taper connections. Did you just couple Ti-Ti and CoCr-CoCr? If so, why not couple CoCr-Ti as is done in many head-neck taper connections?

Thank you very much for this valuable comment. As we mentioned a previous comment, we have simplified the table and described the groups in the text more precisely (L130-140). Due to delivery bottlenecks in CoCr we did not have enough material to also test CoCr-Ti, and we will investigate this material pairing in further investigations. We have added a sentence in this regard to the discussion (L348-350).

Line 180: How was the axial displacement determined (e.g. traverse position, optical measurement…)? I ask because I wonder whether the different inclinations are really caused

by the "stiffness" of the connection as you write in line 318ff or if they reflect rather the deformation of the testing setup with increasing load levels.

Thank you for this important observation. We in fact only recorded the displacement of the actuator of the materials testing machine. The recorded displacement was thus not corrected in the first version of the manuscript. In order to determine the influence of the setup on the measured displacement, we once again carried out tests using only the setup without samples. The upper stamp pressed directly on the lower structure. We determined a stiffness of 39.111 N/mm for the linear range. We then used this value to correct the measured displacement. In the manuscript, we replaced the diagram accordingly and added the following part (L274-275):

"The measured displacement of the testing machine was subsequently corrected for the stiffness of the setup."

We have also added this description to the caption of Figure 6.

Since it might be interesting for the reviewer, we show here the figure of how the graphs have changed after the stiffness correction

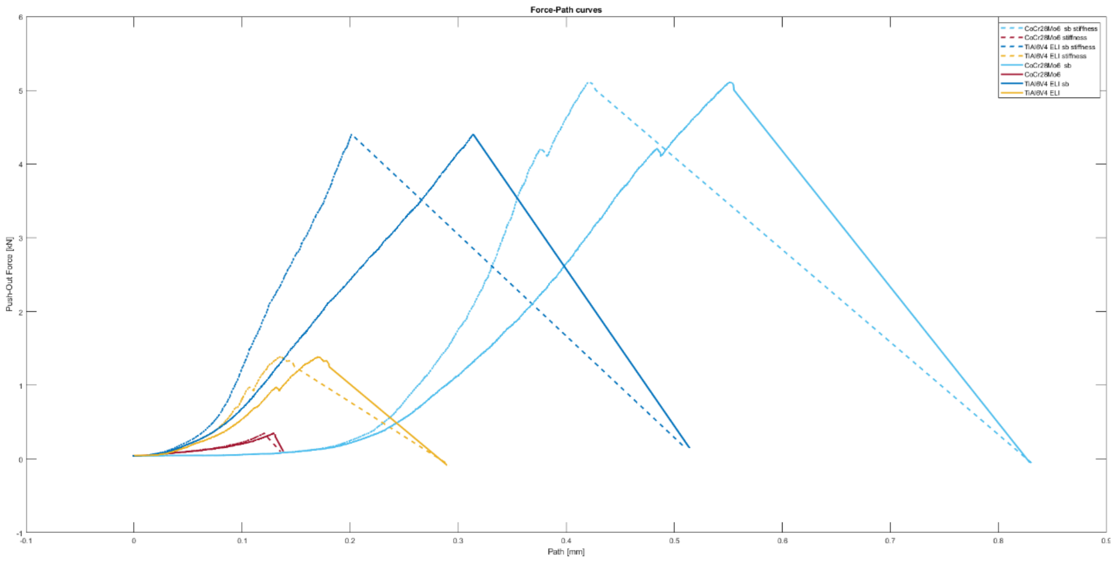

Results

Table 2: Does the table refer to inner or outer tapers?

Thank you very much for this important observation. It was the trunnion, and we have added this to the table heading.

How are the roughness values for the not sandblasted surfaces?

Thank you very much for this comment. We added the values for the not sandblasted surfaces into the table.

Figure 3: Does it refer to inner or outer tapers? Please add the information in the figure caption.

Thank you very much again. Here as above it was the trunnion. We have added this to the figure heading.

The term "Taper mismatch" is used in two different contents, in line 185 ff it refers to the

deviation from the targeted value, later in the paper it refers to the difference between inner and outer taper. This is somehow confusing since "taper mismatch" is more commonly used synonymously to taper clearance (i.e. with respect to the difference between inner and outer taper).

Thank you very much for this important comment. We have adapted the terms throughout the manuscript. We now use 'nominal value' instead of 'target'. And we now only use mismatch to refer to the difference between trunnion and taper.

Line 201-204: The slight differences in taper end diameters do not necessarily result in a changed taper contact situation but different taper angles do (see f.ex. Fig. 8 in Mueller et al., J Arthroplasty 2017, 32(10), 3191). Please use the difference between taper angles in order to characterize the contact (distal or proximal), since using end diameters is very confusing und unusual.

We agree with the reviewer in this regard, we have referred to taper-mismatch to describe the difference in angle between the taper and trunnion and removed the first sentence referring to the end-diameters.

We added the following sentence to the manuscript (L211-214):

"All 28 taper pairs exhibit a negative taper mismatch (trunnion angle < taper angle) and therefore a proximal contact. All taper pairs show a taper mismatch greater than |0.1°| with a mean of -0.182 ± 0.047° (range -0.012° to -0.369°) (table in supplemental material)."

Line 245: Is the correlation statistically significant?

We have added the p-value for the correlations that are significant to the text (L259).

Line 268: If the adhesive is not observed after disconnecting the tapers, where do you think it is gone to? Is it likely that chips of dried adhesive have detached from the surface? If so, this would be a drawback since chips of the glue might act as three-body in the joint articulation or cause tissue reactions. This could be addressed in the discussion.

Thank you very much for this comment. The visual analysis showed that the adhesive adheres only to the sandblasted surfaces and not to the turned ones. So, in the case of sandblasting no chips of glue detached from the sandblasted surface. It will be important to investigate the system under dynamic loading to investigate if particles of dried adhesive are generated and escape into the joint.

Discussion

Line 282f: See comment above (line 201-204). Please refer to the taper angle mismatch if discussing the contact situation.

We agree with the reviewer as above, and therefore we changed the wording and removed the first sentence referring to the end-diameters (L282 first submission of the manuscript).

Reviewer #2: *This manuscript investigates the use of an adhesive and sandblasting on the potential stability of total hip head-neck modular junctions. The study is well-designed. The main criticisms focus on details in the manuscript that overstate the significance of how this study relates to hip modular junctions since the current application is still far removed, and the discussion of previous literature. Most suggestions are minor. In addition, please edit throughout for written English.*

Abstract
The introduction focuses on micromotion as a failure mode. There is a disconnect between these statements and the paper's methods where push-out tests are used. No explanation is given for the disconnect. Please edit accordingly.

Thank you for your comment, in response to this and the authors further comments regarding the introduction, we have significantly edited and reformulated the introduction to address this point.

Introduction
Line 56: citation missing

Thank you very much for the noticing this, we have added an appropriate citation (L55).

Lines 65-66: Reference [3] is an investigation of bi-modular implants which have more than 2 parts, and is less relevant to the current study. Include more references here to make this point, there are plenty of investigations in the literature. Similarly, for the next sentence, the titanium neck adapter was actually TMZF, a different Ti-alloy than what is usually used, so the sentences are misleading.

The literature cited was chosen to generally illustrate the failure of a very promising technology, for hip replacement, bi-modular implants, and our relatively simple study investigating the effect of adhesive bonding on taper junction push-out force. We have attempted to address the reviewers well taken comments on the relevance of the cited literature and our study in significant revisions we have made to the introduction (as suggested by the reviewer below).

Lines 81-82: This is not a relevant finding for the introduction. Taper connections of hip implants on the market have an average angular mismatch much smaller, on the order of 1 minute (.0167 degrees), so 0.096 would already be an outlier and is too large to be clinically relevant.

Thank you for this important comment, which we will attempt to address. It is true that taper mismatch of common stem and head tapers has recently been reported to range from about 0,025’ to -0,14’ (Mueller et al., 2017), which indeed is significantly less than the 0.096° (5.76’) we chose. Nonetheless, positive values that are significantly higher (for example 0.076° to 0.130° or 4.6’ to 7.8’ have also been reported), admittedly for an elongated taper design that is no longer available (Haschke et al., 2016). But more to the point, we have however oriented our study on the possible mismatch constellations that could occur in clinical settings as described by Ashkanfar et al. 2017 and Gustafson et al., 2023, who both conservatively estimated realistic possible taper mismatches based on the literature and their own retrievals to be as high as ±12’. The value we chose, about 6’ falls well within this range.

Suggest an overall re-writing of the literature background about factors that lead to damage in modular THAs. Only a few studies are cited, and they investigate a wide variety of implant types and dimensions that may or may not be relevant to the current study. The paragraphs do not summarize the literature from clinically relevant designs (the bimodular design referenced has been recalled, many studies of angular mismatch reference manufacturing tolerances that, although written in a standard, are much larger than what is actually used by manufacturers to create implants in clinical practice).

Thank you for your suggestion, we have significantly reformulated the introduction to better describe our motivation in relation to the literature. The intention of the introduction was not to provide a comprehensive overview of implant types and dimensions in general. Our motivation is based on the failure of bi-modular implants and their subsequent removal for the market, and our exploratory study of the effect of adhesive bonding on the most rudimentary test of junction stability; the push out test, which to our knowledge has not been investigated to date.

Materials and Methods

Table 1: The labeling of the samples is confusing. Makes it appears that all inner and outer tapers had the same material (Ti-alloy or CoCrMo alloy)? Why would a Ti-alloy taper pair be used when this is not relevant to total hip replacements? Suggest using more descriptive and intuitive labels.

Thank you very much for this important note. We see that the table was quite confusing and therefore we simplified the table and described the groups in the text more precisely.

Results

Lines 192 and 199: I don't know what mismatch means in this context since it is listed for the inner tapers or outer tapers. Difference from the intended value of 5.665? Mismatch usually refers to the difference between inner and outer tapers.

Thank you very much for this important comment. We changed the wording in the section to taper "deviation from nominal value" instead of mismatch (L206-214). And we have added an additional table with the mismatch values to the supplement

The combinations of pairs is very confusing, similar to my comment in the methods. Suggest adding a table that explicitly states which inner taper is paired with which outer taper and the resulting angular mismatch.

Thank you very much for your comment. As written above we simplified the first table and additionally we added another table in the supplemental material with the angular mismatch of every taper pair.

Figure 3: Please use a more descriptive caption. I assume these are all inner tapers because of the process parameters listed.

Thank you very much for the note. We added the missing information in the figure caption.

Even with turning, these would still be considered very smooth inner tapers. Most stem tapers that are turned have trough to peak heights from 10 to 13 microns instead of 3 microns. This is fine, but worthy of a discussion point.

In the basic research oriented approach of this study on the effects of adhesive bonding we chose to investigate the smooth vs. rough surfaces with of a roughness of Rz = 5 µm and Rz = 15 µm respectively. This is admittedly somewhat different than current implants. We have added a sentence to the discussion in this regard (L302-304).

Line 242: Does the free volume take into account all surface differences (angular mismatch, sandblasting, turn machining parameter differences that results in greatly different surface contacts)? It does not seem surprising that there is no correlation between free volume and push-out.

The calculated free volume actually includes the taper mismatch and the roughness profile resulting from the machining process of the trunnion and the taper. We have added a sentence to the discussion in this regard (L250-252).

Lines 256-261: not sure what the purpose of this paragraph is, steeper/not steeper is relevant to a stronger taper pair?

Reviewer#1 raised the question of what influence the stiffness of the test setup has on the results in Figure 6. For this reason, we determined the stiffness of 39,111N/mm for the setup and corrected the data for this.

The gradient of the force-path curves already say something about the stability of the cone connection. the pairings with a greater gradient also tend to have higher force values. And there is less movement in the connection under load.

Discussion
Lines 272-274: what data is this discussion point referring to? It needs to be in the introduction if important.

Thank you very much for this important question. We elaborated this section with more details to make it more understandable (L281-288).

Lines 278-281: Need to discuss the angular mismatch in relation to what is normal for actual THA. Many of the ones in this study would be outliers and not used in clinical practice therefore the conclusions drawn are not relevant/helpful

Thank you for your comment, we have addressed the issue of describing taper-mismatch more clearly in response to a comment from the first reviewer. We now report the taper mismatch of the specimens tested (L211-214 and table in supplemental material), and that these did not significantly affect push out-force in the passage of the discussion the reviewer was referring to (L281-288).

Lines 306-308: What does suitable mean in this context? What about the other studies in the literature? There have been EXTENSIVE studies with pull out/push out etc. The conversation about "suitable" is irrelevant to the paper. Recommend only saying that sandblasted surfaces resulted in the highest push-out forces that were greater than 2 kN.

Thank you for insight, you are of course correct. We have reformulated the paragraph and added the relevant literature. Our goal was however not to show that adhesively bonded tapers are better than any taper ever tested, but that in the system we investigated bonding results in significant increases of push-out force.

The limitations section is not extensive enough. (lines 329-330). What are the more realistic conditions? What about realistic taper mismatch, etc?

Thank you very much for this valuable comment. We extended the limitation section with further points and information (L344-350). Please also see comment on L278-281.

Conclusion
Line 334-335: Please remove "generally accepted". There is no generally accepted value in this field.

Thank you very much for this observation. We have revised the sentence as follows:

“The push-out forces generated from the sandblasted tapers resulted in the highest push-out forces and were greater than push out forces from mechanical assembled taper pairs reported in the literature.”

All figures and tables: use better abbreviations or spell out entire words. Figures and tables are difficult to understand

Thank you very much for this important note. We changed the figure captions of figure 3,5 and 6 as well as the tables 1 and 2 to make them more understandable.
