# Supplementary material for: Influence of the use of an adhesive connection on the joint strength of modular hip endoprostheses
Source: PLoS One. 2024 Nov 18;19(11):e0313964. doi: 10.1371/journal.pone.0313964 (PMC11573162; doi:10.1371/journal.pone.0313964)
Supplement: Attachment — Submitted filename: Response to ReviewersR2.docx [file pone.0313964.s007.docx]

Dear Dr. Antonio Riveiro Rodríguez,

Thanks once again for the opportunity to revise our paper in order to resubmit it to PLOS ONE.

We thank the reviewers for their work and have revised the manuscript and Figure 4 in accordance with the comments.

***Reviewer #1:*** *The authors have answered all comments satisfactorily. I have one more remark regarding Figure 4, please use the taper terms used in the text also for the figure (taper and trunnion instead of inner taper and outer taper). The manuscript can be accepted for publication now.*

Enclosed is the modified figure 4:

***Reviewer #3:*** *Please recheck the style of references. Did you use the Numbered or Vancouver?*

Thank you for pointing this out, we apologize for this inadvertence. We have adjusted the references and citations in accordance with the journal's specifications.

***Reviewer #3:*** *Please write your hypothesis here.*

On recommendation, we have added the following sentence at the end of the introduction (L108-109):

“We hypothesize that a bonded connection with a rough surface achieves at least the same strength as conventionally joined implant junctions.”

***Reviewer #3:*** *Please check references 11 and 13. No pages are listed for these articles.*

Many thanks for the hint. We have added the information for the page numbers
